# Promising Therapeutic Strategies for Hematologic Malignancies: Innovations and Potential

**DOI:** 10.3390/molecules29174280

**Published:** 2024-09-09

**Authors:** Jan Jakub Lica, Bhaskar Pradhan, Kawthar Safi, Joanna Jakóbkiewicz-Banecka, Andrzej Hellmann

**Affiliations:** 1 Faculty of Health Science, Powiśle University, 80-214 Gdańsk, Poland; 2 Department of Biochemistry, Faculty of Pharmacy, Medical University of Warsaw, 02-097 Warsaw, Poland; bhaskar.pradhan@wum.edu.pl; 3 Department of Biochemistry and Clinical Chemistry, Faculty of Biology, Medical University of Warsaw, 02-097 Warsaw, Poland; 4 Department Medical Biology and Genetics, Faculty of Biology, University of Gdańsk, 80-308 Gdańsk, Poland; 5 Department of Hematology and Transplantology, Faculty of Medicine, Medical University of Gdańsk, 80-214 Gdańsk, Poland; andrzej.hellmann@gumed.edu.pl

**Keywords:** hematologic cancers, innovative approaches, therapeutic modalities, synergistic potential, PI3K inhibitors, proteasome inhibitors, NF-κB inhibitors, immunotherapy checkpoint inhibitors, neddylation inhibitors, small molecules

## Abstract

In this review we explore innovative approaches in the treatment of hematologic cancers by combining various therapeutic modalities. We discuss the synergistic potential of combining inhibitors targeting different cellular pathways with immunotherapies, molecular therapies, and hormonal therapies. Examples include combining PI3K inhibitors with proteasome inhibitors, NF-κB inhibitors with immunotherapy checkpoint inhibitors, and neddylation inhibitors with therapies targeting the tumor microenvironment. Additionally, we discuss the potential use of small molecules and peptide inhibitors in hematologic cancer treatment. These multidimensional therapeutic combinations present promising strategies for enhancing treatment efficacy and overcoming resistance mechanisms. However, further clinical research is required to validate their effectiveness and safety profiles in hematologic cancer patients.

## 1. Introduction

Hematologic malignancies (HMs), encompassing a diverse array of cancers affecting blood, bone marrow, and lymph nodes, present a significant clinical challenge due to their heterogeneity and propensity for treatment resistance [1,2]. In 2020, the global incidence of leukemia was approximately 474,519 new cases, according to GLOBOCAN data from the International Agency for Research on Cancer (IARC). This represents about 2.6% of all new cancer cases worldwide [3]. NHL had an estimated 544,352 new cases in 2020, while HL accounted for about 83,087 new cases [4]. Despite advances in therapy, achieving durable remissions remains elusive in many cases. Traditional treatments for hematologic malignancies include chemotherapy, radiotherapy, stem cell transplantation (SCT), and therapeutic antibodies [5,6]. However, these approaches often lead to adverse effects such as organ damage, fatigue, nausea, cytokine syndrome, cardiotoxicity, and autoimmune reactions [7,8]. Moreover, the diversity and unique characteristics of these malignancies necessitate tailored treatment approaches. For instance, contemporary therapy for leukemia often involves a combination of therapeutic antibodies like fludarabine-cyclophosphamide-rituximab, Pi3k inhibitor, and Venetoclax [9,10]. In contrast, Multiple Myeloma (MM) treatments are increasingly combining immunotherapy with monoclonal antibodies and Chimeric Antigen Receptor T-cell (CAR-T) therapies [9], while lymphoma treatment predominantly relies on classical approaches such as Rituximab, Etoposide, and multidrug chemotherapy [11]. 

Multimodal therapeutic strategies have emerged as a promising avenue to overcome these challenges, leveraging the synergistic effects of various treatment modalities to enhance efficacy and overcome resistance mechanisms. Recent advancements in therapeutic strategies for hematologic malignancies include the development of innovative immunotherapies such as bispecific CAR-T therapy, bispecific killer cell engagers, trispecific killer cell engagers, and dual affinity retargeting therapies [12]. Additionally, exosomes have emerged as promising cell-free tumor treatment alternatives, capable of delivering drugs, genes, and therapeutic substances to modify the tumor microenvironment [13]. Furthermore, antibody-based immunotherapies like cancer vaccines, oncolytic virus therapies, monoclonal antibody treatments, and CAR-T cell therapies have shown longer survival times and fewer adverse reactions compared to conventional treatments, emphasizing the potential of immunotherapy in treating hematologic malignancies [14]. 

These innovative approaches signify a shift towards more effective and targeted treatment modalities, offering new hope for improved patient outcomes and reduced complications. This review aims to explore the potential of these strategies, including immune checkpoint inhibitors, PI3K inhibitors, TIM-3 and TIGIT inhibitors, neddylation inhibitors, and CAR-T cell therapies.

## 2. Therapeutic Strategies

### 2.1. Immune Checkpoint Inhibitors

Immune checkpoint inhibitors (ICIs) have shown significant success in treating solid tumors, but their efficacy in hematologic malignancies remain under investigation. ICIs work by eliciting T-lymphocyte-mediated anti-tumor responses. Currently, FDA-approved ICIs such as Nivolumab and Pembrolizumab are used for treating classic Hodgkin lymphoma and primary mediastinal B cell lymphoma [15]. Multiple clinical trials are ongoing, exploring drug candidates such as PD-1/PDL-1 and CTLA-4 inhibitors [16]. Other T-lymphocyte checkpoints, including LAG-3, TIM-3, TIGIT are also being studied with a focus on long term safety and efficacy [17]. 

TIM-3 (T cell immunoglobulin and mucin domain 3) is an inhibitory immune checkpoint receptor co-expressed with PD-1 on tumor-infiltrating immune cells, including dendritic cells and NK cells (Table 1). Its inhibition leads to T cell exhaustion and apoptosis. TIM-3 ligands include Galectin-9 (GAL-9) and carcinoembryonic antigen-related cell adhesion molecule (CEACAM1). Interestingly, it has been postulated that TIM-3 signaling plays a role in PD-1 directed therapy resistance due to the co-expression of both receptors, making it an attractive target for investigation [18].

TIGIT (T cell immunoglobulin and ITIM domain) is another potential target involved in T cell inhibition and immune escape in hematological malignancies (Table 2). TIGIT is expressed on T cells, regulatory T cells, and NK cells, and activates an immunoregulatory network on antigen-presenting and cancer cells. Its primary ligand is CD155 [19,20], leading to T cell inhibition through receptor downregulation and competition with the activating co-receptor CD226. By targeting TIGIT, inhibitors disrupt its immunosuppressive signaling pathways, aiming to enhance the activity of tumor-infiltrating T cells and improve anti-tumor immune responses. These inhibitors represent promising approaches to counteract TIGIT-mediated immune suppression and enhance anti-tumor immunity in cancer patients [21].

### 2.2. Small Molecule Inhibitors 

Small molecules (with molecular weight under 1 kDa), such as peptides, remain an attractive option for treating hematological cancers. For example, the dipeptide melphalan is used for MM. These molecules promote DNA damage, triggering apoptosis in cancer cells. Other peptides, like proteasome inhibitors, such as borteozomib, have high lipophilicity, enabling them to penetrate the skin easily and cross the blood–brain barrier. Proteasome inhibitors like boratezomib play a crucial role in maintaining cellular homeostasis by regulating the turnover of cellular proteins. Bortezomib binds reversibly to the chymotrypsin-like subunit of the 26S proteasome, inhibiting its activity and preventing the degradation of several pro-apoptotic components. This inhibition leads to the accumulation of pro-apoptotic proteins, which in turn triggers programmed cell death via caspase-mediated pathways, particularly in neoplastic cells that rely on the suppression of pro-apoptotic pathways for their proliferation and survival [22]. 

Ubiquitin-Specific Proteases 7 (USP7) belong to the USP family of cysteine proteases that are actively researched in the context of deubiquitinating enzymes (DUBs). Many proteins, including p53, MDM2, BRCA1-A, p21, and beta-catenin, that are implicated in the pathways leading to the advancement of cancer, are deubiquitinated by USP7 [23]. P5091, an inhibitor of USP7, induces apoptosis in MM cells that are resistant to bortezomib and conventional treatments. Biochemical and genetic studies demonstrate that P5091-induced cytotoxicity is mitigated by inhibiting HDM2 and p21. In vivo studies indicate that P5091 is well tolerated, inhibits malignant cell growth, and extends survival [23]. Another molecule, known as NCT02372240 or VLX1570, inhibits USP14 but has shown severe pulmonary toxicity in preclinical studies [19]. Utilizing ubiquitin-like proteins (ULPs) as an alternative pathway for protein degradation has also unveiled new targets for cancer therapy. ULPs contribute to approximately 20% of protein degradation through the proteasome system.

Modern approaches utilizing small molecules, especially those with molecular weight under 1 kDa, are gaining fresh impetus in the treatment of such malignancies (Table 3). The molecules offer advantages such as easier cellular entry, oral effectiveness, and comparatively cost-efficient synthesis [23].

### 2.3. PI3K Inhibitors

**Figure 1:**
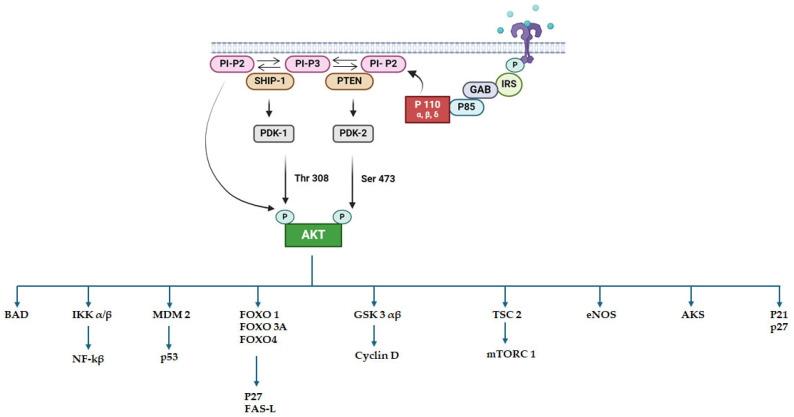
PIK3/AKT/mTOR pathway involved in tumorigenesis and cancer progression pathway. Adaptors like Gab2 or IRS family proteins are drawn to the regulatory p85 subunit of PI3K by an active tyrosine kinase receptor (RTK). This eventually activates the catalytic p110 abc subunits of PI3K. Activated PI3K complex transforms PI-P2 into PI-P3. The latter recruits PDK1 and AKT to the plasma membrane where AKT is phosphorylated by PDK1 on Thr308. PDK2, which is mTORC2, phosphorylates AKT on Ser473. This activated AKT modulates several substrates which are necessary for cell survival, the cell cycle, and cell growth.

The PI3K/Akt/mTOR is an essential pathway for regulating cell growth and proliferation. Activation of this pathway is critical for leukemogenesis and is linked to a poor prognosis (Figure 1). Several molecular abnormalities lead to the activation of this pathway, with its activation varying based on the behavior of hematological malignancies. Diverse HMs activate this pathway through genetic mutations, including the oncogenes phosphatidylinositol-4,5-bisphosphate 3-kinase catalytic subunit alpha (*PIK3CA*) and phosphoinositide-3-kinase regulatory subunit 1 (*PIK3R1*), and the tumor suppressor gene phosphatase and tensin homolog (*PTEN*) [20]. Since PTEN is a major regulating pathway of PI3K/Akt/mTOR signaling, loss of *PTEN* contributes to the upregulation of this signaling cascade, eventually resulting in enhanced cell proliferation and chemoresistance in AML, CML, and ALL. Apart from *PTEN* mutations, *FLT3* mutations induce proliferation in AML through mTOR signaling, whereas in CML, BCR-ABL kinase binds to the p85 PI3K regulatory subunit, thereby activating the PI3K/Akt/mTOR pathway [24]. Therefore, inhibition of PI3K might generate novel therapeutic promise against HMs (Figure 1).

Currently approved inhibitors of PI3K include idelalisib, duvelisib, and umbralisib. While these are commonly used for the treatment of relapsed/refractory (RR) HMs, their mechanism of action and targets are diverse. Preclinical studies have demonstrated that idelalisib exerts dose-dependent cytotoxicity by induction of caspase-dependent apoptosis in Chronic Lymphocytic Leukemia (CLL) patients [25]. Moreover, reduction in AKT and mitogen-activated protein kinase (MAPK) activity have been observed in CLL cells treated with idelalisib. Idelalisib also affects the tumor microenvironment by preventing the secretion of cytokines and chemokines, it interferes with the connection between CLL cells and monocyte-derived nurse-like cells (NLCs), and it eventually reduces chemotaxis [26].

Regarding duvelisib, the predominant expression of PI3Kγ is on myeloid and T cells, while PI3Kδ is mostly seen on leukocytes. Therefore, PI3K inhibition aims to lower cytokine synthesis that promotes leukemic cell survival. In preclinical investigations, duvelisib exposure resulted in direct cytotoxicity due to lowered production of pro-survival cytokines in leukemic B cells [27]. In comparison to blocking either isoform alone, duvelisib was found to be more effective at dual PI3K inhibition in animal models [28].

Umbralisib is considered a next-generation, highly selective PI3Kδ inhibitor that also inhibits casein kinase-1ε (CK-1ε), a protein implicated in the regulation of the Wnt5a pathway and the translation of the *c-Myc* oncogene. In silico docking experiments have revealed that umbralisib binds to, and inhibits, the catalytic site of CK1ε through the core pyrazolopyrimidine amine moiety. Furthermore, two forms of kinome profiling verified the specificity of umbralisib for only PI3Kδ and CK1ε, with no off-target inhibition [29,30]. This dual mechanism of action allows Umbralisib to exert its effects on hematological malignancies by targeting both PI3Kδ and CK1e pathways simultaneously, potentially leading to enhanced therapeutic outcomes in patients with relapsed or refractory marginal zone lymphoma and follicular lymphoma [30]. In vitro, umbralisib tends to inhibit malignant cell proliferation, CXCL12-mediated cell adhesion, and CCL19-mediated cell migration [31]. (For the overview of PI3K inhibitors see Table 4 below).

**Figure 2:**
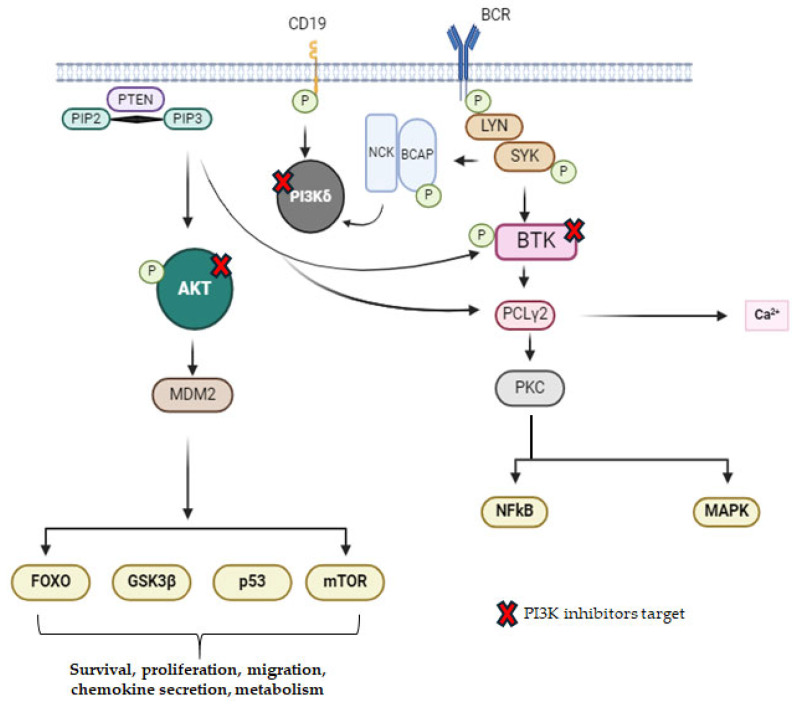
Schematic representation of PI3K pathway with inhibitors target.

Despite the approval of these inhibitors by FDA for some HMs, questions regarding their efficacy and adverse effects (AE) remain. Therefore, some novel small molecule inhibitors of PI3K are currently in early phase clinical trials. These include Zandelisib (ME-401), Linperlisib, TQB3525, Acalisib, and SHC014748M [32,33] (Figure 2).

Additionally, in vivo studies have concluded that the co-blockage of CEACAM1 and TIM-3 leads to the enhancement of the anti-tumor immune responses in colon cancer. With regard to other neoplasms, Tim-3 also plays a significant role in the development and progression of gastric cancer [34]. Its expression levels on CD4+ T cells are a deciding factor for clinicopathological parameters such as tumor size, lymph node involvement, and depth of tumor invasion. Combined Gal-9/Tim-3 signaling can promote the secretion of IL-6, IL-8, and IL-10 from monocytes, which has been found to be correlated with poor treatment response [35]. Currently researched TIM-3 inhibitors are being developed for cancer immunotherapy. The idea behind these drug candidates is to provide potential therapies to overcome immune suppression and enhance anti-tumor immune responses in various cancers. Antibodies against TIM-3 also utilize the pharmacological inhibition of the MAPK pathway. Experiments with T cell-deficient mice revealed that an extent of reversal of Tim-3 Ab-induced tumorigenesis occurred. Upon blockage of TIM-3, it was noted that MAPK pathway proliferative mediators were prevented from phosphorylation, thereby enhancing anti-tumor activity [34]. 

### 2.4. Targeting the NF-κB Pathway

Hematopoietic stem cells’ (HSCs) self-renewal and differentiation into myeloid and lymphoid lineages are regulated by NF-κB, a critical biological regulator that controls a variety of processes including cell survival, apoptosis, invasion, and hematopoiesis [36]. The NF-κB family comprises five members: p65 (RelA), RelB, Rel (c-Rel), and the precursor proteins NF-κB1 (p105) and NF-κB2 (p100), which undergo processing to become their active forms, p50 and p52, respectively. These transcription factors typically function as homodimers or heterodimers, and translocate to the nucleus where they bind to non-canonical sequences or classical κB sites to either stimulate or repress gene expression. The most prevalent active NF-κB complex in mammalian cells is the p65/p50 heterodimer [37]. Under normal physiological conditions, NF-κB interacts with NF-κB inhibitory proteins (IκBs) to remain inactive in the cytoplasm [38].

**Figure 3:**
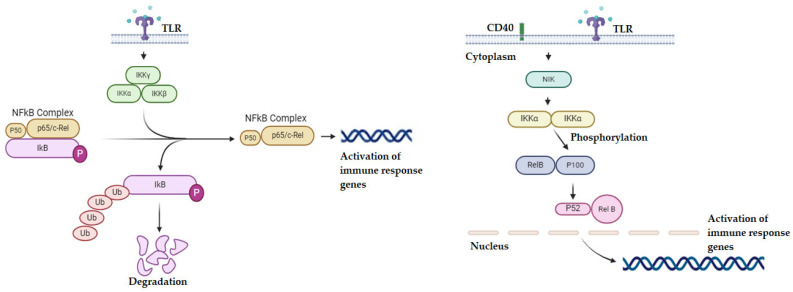
**Left panel**: The illustration depicts the canonical and non-canonical NF-κB pathways on the left and right, respectively. A variety of ligands, including growth factors, tumor necrosis factor (TNFα), and Toll-like receptors (TLRs), mediate the activation of the canonical route. The process of activation is dependent on the IKK complex phosphorylating IκB-α and the proteasome, then degrading it. As a result, the Rel/p50 complex moves into the nucleus and starts to transcriptionally regulate the target genes. **Right panel**: schematic representation of non-canonical NF-κB pathways. Canonical NF-κB pathway: stimuli (e.g., B cell and T cell receptors, TNFR), IKK complex activation (phosphorylates IκBα), IκBα degradation (releases NF-κB dimers), NF-κB dimer translocation (p50/RelA, p50/c-Rel), and gene regulation (stimulates or represses expression).

The activation of NF-κB involves two distinct pathways: the canonical and the non-canonical (alternative) (Figure 3) pathways. Both pathways are crucial for controlling inflammatory and immunological responses [39]. The canonical NF-κB pathway responds to a wide range of stimuli, such as B-cell and T-cell receptors, TNF receptor (TNFR) superfamily members, pattern-recognition receptors (PRRs), and ligands of other cytokine receptors. The primary process of canonical NF-κB activation occurs via the inducible degradation of NF-κB which is initiated by the site-specific phosphorylation by the IκB kinase (IKK) complex [40]. Upon activation, the IKK complex phosphorylates IκBα at two N-terminal serines, leading to its ubiquitin-dependent degradation in the proteasome. This degradation releases the NF-κB dimers, primarily p50/RelA and p50/c-Rel, which then translocate to the nucleus to regulate gene expression [41].

The non-canonical NF-κB pathway reacts preferentially to a specific set of stimuli, such as ligands of a certain TNFR superfamily member, including LTβR, BAFFR, CD40, and RANK. Unlike the canonical NF-κB pathway, which relies on IκBα degradation, non-canonical NF-κB activation depends on the processing of the NF-κB2 precursor protein, p100 [42]. NF-κB-inducing kinase (NIK) is a key signaling protein in this pathway, functioning alongside IKKα to promote p100 phosphorylation. This phosphorylation triggers p100 ubiquitination and subsequent processing [43]. Degradation of the C-terminal IκB-like structure of p100 leads to the maturation of NF-κB2 p52 and nuclear translocation of the non-canonical NF-κB complex p52/RelB [44].

Modulation of NF-κB signaling cascades can occur at various stages, including gene transcription, post-translation, IKK complex activation, or any intermediate stage. Small compounds, peptides, oligonucleotides, monoclonal antibodies (mAbs), and small interfering RNA (siRNA) have been utilized to achieve this modulation [45]. Other small molecules targeting NF-κB activation include Bay 11-7082 and resveratrol. Bay 11-7082 inhibits NF-κB activation by targeting the IκB kinase complex, preventing the phosphorylation and subsequent degradation of IκBα. Resveratrol suppresses the phosphorylation and degradation of IκBα, thereby preventing the translocation of NF-κB to the nucleus and inhibiting its activation [46,47]. (For the overview of inhibitors targeting the NF-κB pathway see Table 5 below).

### 2.5. CD47 Inhibitors 

CD47, also known as cluster differentiation 47, is found in abundance on the surface of cells. Recently, the role of CD47 as a modulator of innate immune surveillance has gained significant attention, particularly when it interacts with the membrane protein SIRPα (SHPS-1/BIT/CD172a) on macrophages and other myeloid cells [48]. Increasing evidence suggests that disrupting the association between CD47 and SIRPα can enhance the ability of macrophages to eliminate malignant cells [49].

**Figure 4:**
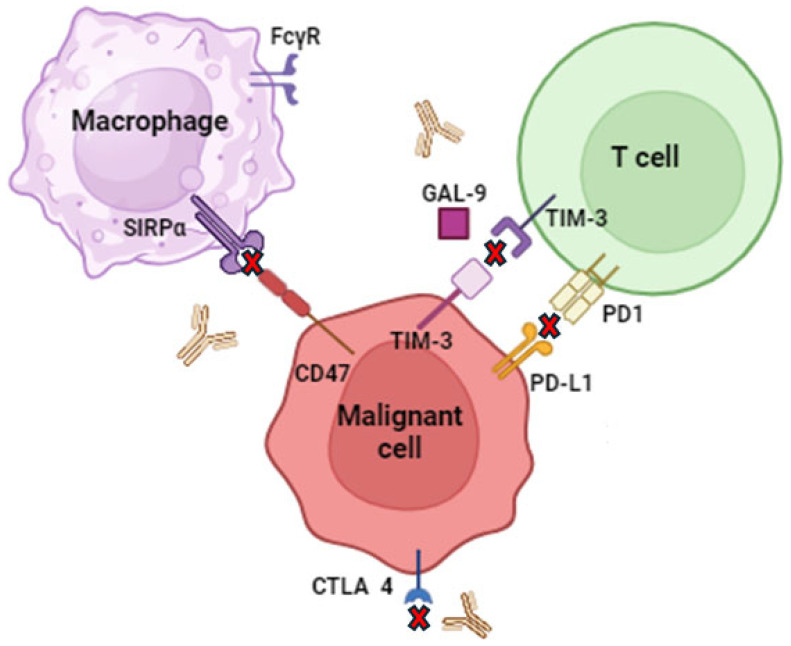
Schematic representation of CD47 inhibitors’ target. The red ‘x’ represents inhibition.

In the context of hematologic malignancies, such as leukemia and lymphoma, CD47 plays a crucial role in protecting cancer cells from being phagocytosed by macrophages (Figure 4). Tumor cells often overexpress CD47, which sends a “don’t eat me” signal to macrophages via its interaction with SIRPα. By blocking CD47, this protective signal can be disrupted, making cancer cells more susceptible to immune-mediated destruction. This mechanism is particularly relevant in hematologic cancers, where the tumor cells are in direct contact with immune cells in the blood and bone marrow microenvironments [50].

Clinical trials have shown that targeting CD47 can be an effective therapeutic strategy in treating hematologic malignancies. For example, Hu5F9-G4, a monoclonal antibody against CD47, has demonstrated promising results in early-phase clinical trials for the treatment of Acute Myeloid Leukemia (AML) and non-Hodgkin lymphoma. These trials, presented in Table 6, have reported increased phagocytosis of cancer cells and reduction in tumor burden [51].

Moreover, combining CD47 blockade with other treatments, such as chemotherapy, monoclonal antibodies (e.g., rituximab for B-cell lymphomas), or immune checkpoint inhibitors (e.g., PD-1/PD-L1 inhibitors), can further enhance anti-tumor efficacy. The combination therapies aim to synergistically boost the immune system’s ability to target and eliminate cancer cells, while also overcoming resistance mechanisms that tumors may develop [52]. 

### 2.6. Neddylation Inhibitors

Neddylation inhibitors, such as MLN4924 and TAS4464, target the NEDD8-activating enzyme (NAE) to disrupt the neddylation pathway, a post-translational modification crucial in regulating protein functions and homeostasis [53,54]. NEDD8 (neural precursor cell expressed developmentally downregulated-8) is an 81-amino acid peptide similar to ubiquitin. In hematological malignancies like AML, overactivation of neddylation, indicated by upregulated NEDD8, UBA3, UBE2M, and RBX1, correlates with poor patient outcomes [54]. Inhibition of neddylation by MLN4924 triggers anti-leukemia effects by inducing cell apoptosis, senescence, and autophagy, while activating the p53 signaling pathway, thus serving as a potential therapeutic strategy in AML treatment [54]. Additionally, neddylation inhibition impacts the tumor microenvironment, influencing immune cells and other components crucial for tumorigenesis, highlighting the broader anticancer efficacy of targeting neddylation in hematological malignancies [55].

It functions through NAE, which catalyzes the linkage of NEDD8 to lysine residues on target proteins, a process known as neddylation. Overexpression of neddylation-related proteins, such as NEDD8, UBA3, UBE2M, and RBX1, is associated with tumor progression and poor prognosis in hematological malignancies like MM and Myelodysplastic Syndromes [56].

Inhibition of neddylation by MLN4924 triggers anti-leukemia effects by inducing cell apoptosis, senescence, and autophagy while activating the p53 signaling pathway [54]. This inhibition impacts the tumor microenvironment, influencing immune cells and other components crucial for tumorigenesis, highlighting the broader anticancer efficacy of targeting neddylation in hematological malignancies. Additionally, TAS4464, a highly potent NAE inhibitor, selectively inhibits NAE, inducing cullin neddylation inhibition and the accumulation of CRL substrates. This results in widespread antiproliferative activity in cancer cell lines and patient-derived tumor cells, making it a promising agent for hematologic tumors [57].

Combining neddylation inhibitors with molecular therapy to target the tumor microenvironment, as well as with hormonal therapy, immunotherapy-based biological therapy, and CD47 receptor blockade (“don’t eat me” action) may enhance treatment efficacy. This combination could stimulate cancer cell phagocytosis by macrophages and eliminate chemo and immunotherapy resistance [50]. Neddylation inhibitors, thus, represent promising candidates for cancer therapy by targeting the ubiquitin–proteasome system and disrupting the proteostasis of cancer cells. Ongoing preclinical and clinical studies aim to elucidate their efficacy and safety profiles in various cancer types [58]. 

**Figure 5:**
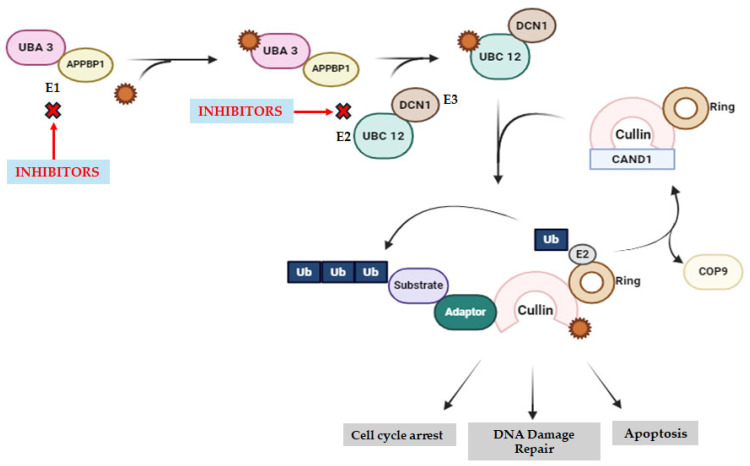
Neddylation inhibition targeting sites. It blocks enzymatic activity in the NAE active site by creating a covalent NEDD8-PEV adduct, which inhibits NEDDylation. A subset of Cullin-RING ligases are prevented from becoming NEDDylated by DI-591 and NAcM-OPT, which interfere with DCN1’s ability to bind to UBC12. Abbreviations: NAE—NEDD8 activating enzyme; UBA3—ubiquitin-like modifier activating enzyme 3; APPBP1—NEDD8 activating enzyme E1 regulatory subunit; UBC12—NEDD8 conjugating enzyme Ubc12; DCN1—defective in cullin NEDDylation 1; Ub—ubiquitin; COP9—constitutive photomorphogenesis 9; CAND1—cullin-associated NEDD8-dissociated protein 1.

Further research into these neddylation inhibitors and related compounds holds promise for the development of novel cancer therapeutics targeting the ubiquitin–proteasome system. Preclinical and clinical studies, represented in Table 7, are underway to evaluate their efficacy, safety, and therapeutic potential in cancer treatment. Overall, compounds impeding neddylation, such as pevonedistat (MLN4924) and TAS4464, could be valuable in treating hematologic cancers by governing and interfering with the physiological protein degradation processes [59] (Figure 5 and Table 7).

### 2.7. PD-1 Inhibitors

PD-1 (programmed cell death protein 1) is a common immunosuppressive component found on the surface of T cells. It is essential for promoting self-tolerance and suppressing the immune system. On the surface of malignant tumor cells, its ligand programmed cell death ligand 1 and 2 (PDL-1 and PDL-2) is overexpressed. The binding of PD-1 to its ligands slows the growth of PD1-positive cells and aids in the immune evasion of malignancies, resulting in treatment failure [60]. These PD-1 inhibitors have revolutionized the treatment of various cancers and have demonstrated durable responses and improved survival outcomes in patients with advanced or metastatic disease [61]. They are often used as monotherapy or in combination with other anticancer therapies (Table 8), such as chemotherapy, targeted therapy, or other immunotherapy agents (Figure 4).

Although, PD-1/PD-L1 inhibitors exhibit potent anti-tumor activity, most patients could not benefit from this treatment, resulting in primary or acquired treatment resistance. In recent years, combining PD1 therapy with other treatments has been considered a rational and most feasible approach [62]. The PD1/PDL1 interaction is inhibited by monoclonal antibodies (mAb), also referred to as checkpoint inhibitors, which alleviate the drawbacks of conventional anticancer therapy (Figure 4). Lussier et al. have discovered that T cell function can be improved by inhibiting PD1 using antibodies based on both in vitro and in vivo experiments. Monoclonal antibodies have the potential to greatly reduce toxicity, decrease the size of solid tumors, block advanced cancers and metastases, and increase patient survival rates [63].

Monoclonal antibodies (MA) have significantly advanced the treatment of hematological malignancies, providing targeted therapies that reduce toxicity, enhance efficacy, and improve patient outcomes. The continued development of MA, including newer antibody-drug conjugates and bispecific antibodies, holds promise for even more effective treatments in the future and are presented in Table 9.

### 2.8. CTL-4 Inhibitors

CTL-4 (cytotoxic T-lymphocyte-associated antigen 4) is considered one of the most important immune checkpoint receptors. As a class of inhibitory receptors, immunological checkpoints are essential for controlling effector immune cells, preventing them from eradicating healthy cells and triggering autoimmune disorders. However, malignant cells exploit this mechanism to evade the immune system and suppress the effector activities, which can lead to immunosurveillance. Therefore, blocking CTLA-4 can help control immune escape and promote the anti-tumor activity [70]. 

The CTLA-4 blockade influences the immune priming phase by promoting the activation and proliferation of a greater number of effector T cells, independent of TCR specificity, and by diminishing the Treg-mediated suppression of T cell responses [71]. These inhibitors target CTLA-4, a key regulator of T cell activation, and have shown promise in enhancing the immune response against cancer cells. They are being studied either as a monotherapy or in combination with other immunotherapies in various clinical settings (Table 10).

### 2.9. T Cell and NK-Cell Therapy

**Figure 6:**
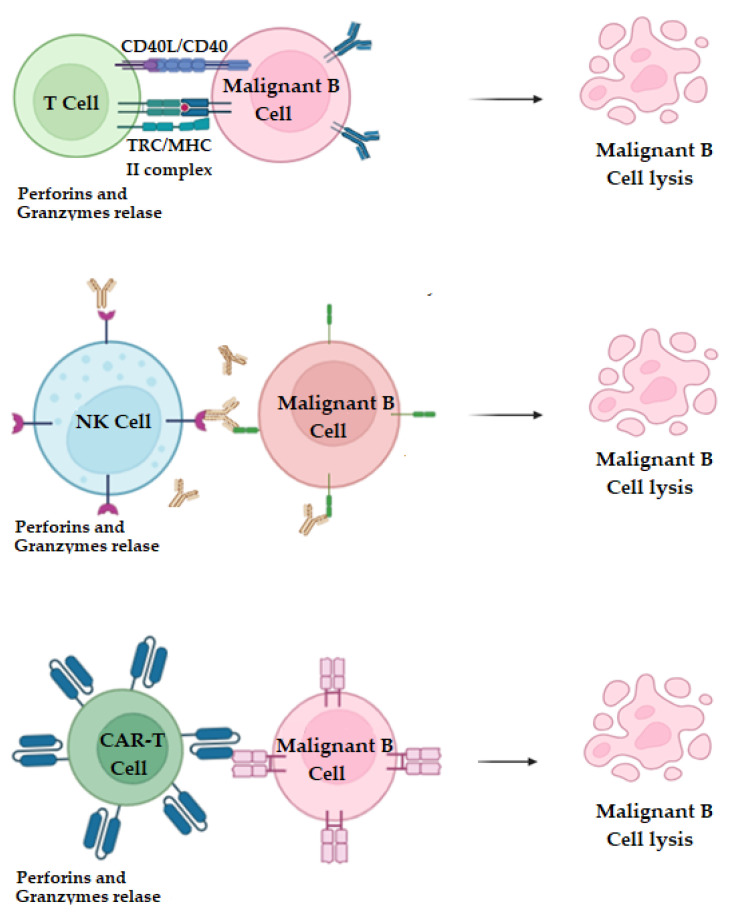
**Top Panel**: ligand receptor recognition therapy; under NK and T cell therapies. Target cell missing-self recognition. The activity of natural killer (NK) cells is controlled by the signaling from activating and inhibitory receptors. MHC class I molecules, a ligand for NK cell inhibitory receptors, are lost by stressed cells, such as tumor cells. Simultaneously, they obtain chemicals linked to stress, serving as ligands to stimulate receptors. Therefore, the balance is shifted toward NK cell activation due to the lack of inhibitory signaling and the stimulation of activating signaling, which results in the production of cytokines and the death of tumor cells. **Middle Panel**: natural killer (NK) cells and other leukocytes participate in antibody-dependent cell-mediated cytotoxicity (ADCC) by binding to antibody-coated target cells through their Fc receptors, leading to the destruction of the target cells. **Bottom Panel**: chimeric antigen receptors or CARs are genetically engineered T cells, isolated from the patient, which are modified to recognize the target surface antigens and degrade them by releasing cytokines like perforin and granzymes.

Immunotherapies based on NK (natural killer) and T cells are gaining fresh impetus in contemporary treatment methods (Figure 6). Due to their unique ability to recognize and eliminate target cells without antigen-specific activation, they are of significant interest to researchers [72]. Recent improvements using these cells have revealed their significant therapeutic potential as both a combined therapy and a monotherapy. Additionally, a variety of novel approaches have been developed, drawing inspiration from the properties of NK and T cells. These approaches include the use of NK or T cells in combination with immune checkpoint blockade, chimeric antigen receptor (CAR)-expressing NK or T cell treatment, and artificial adjuvant vector cells [73]. Some of these approaches are listed in Table 11. 

### 2.10. Macrophages

Macrophages are known to be the most important effector cells of the innate immune system. The activation of macrophages depends upon their location and specific microenvironmental stimuli and signaling. Their fate can be determined based on these events, classifying them as either classically activated (M1) or alternatively activated (M2) polarization types [74]. M1 macrophages are pro-inflammatory and tumor-inhibiting, whereas M2 macrophages are anti-inflammatory and tumor-supporting. Macrophages which infiltrate the tumor microenvironment (TME) are referred to as tumor-associated macrophages (TAMs) [75]. 

Specifically, CSF-1 and IL-10 are two tumor-derived molecules that encourage a significant percentage of TAMs to develop into M2 macrophages [76]. While TAMs can contribute to tumor surveillance and eradication, recent research reveals that they may paradoxically play a pivotal role in tumorigenesis by encouraging angiogenesis, metastasis, cancer stemness, and local immunosuppression within the TME, thus contributing to neoplastic progression [77].

Recent research highlights the crucial role of TAMs in hematological malignancies. In hematological malignancies such as leukemia, lymphoma, and myeloma, macrophages infiltrate the disease microenvironment, develop specific activation attributes, and contribute to disease progression. Macrophages in the leukemic microenvironment is referred to as leukemic-associated macrophages (LAMs) [78]. These malignancies exhibit defense mechanisms against the immune system, and, by comprehending these mechanisms, novel strategies to trigger the immune system to perceive cancer as alien can be developed [78]. 

Over the past decade, rigorous efforts have been made to target checkpoint blockade immunotherapy by expressing inhibitory receptors, which include PD-L1, SIRPα, CTLA-4, ultimately resulting in the activation of immune response [79]. These strategies utilize the activation of macrophages as a promising agent towards hematological malignancies (Figure 4) (Table 12).

Macrophages are a double-edged sword in cancer. Tumor-associated macrophages (TAMs) can either support tumor growth by promoting angiogenesis, suppressing the immune response and aiding metastasis, or they can help fight tumors by presenting antigens and activating cytotoxic T cells. Therapeutic strategies are being developed to reprogram TAMs from a tumor-promoting (M2) phenotype to a tumor-fighting (M1) phenotype. Approaches include the use of cytokines, small molecules, and antibodies to modulate macrophage function [80]. Macrophages play a key role in tissue repair and regeneration. They can promote the healing process by clearing dead cells and debris, and by releasing growth factors that stimulate tissue repair. Therapies that harness macrophages are being explored to improve wound healing and treat chronic wounds [81]. In autoimmune diseases, these cells can contribute to tissue damage by producing inflammatory cytokines. Therapies targeting macrophages aim to reduce inflammation and tissue destruction by inhibiting the activation of pro-inflammatory macrophages [82].

Chimeric antigen receptor macrophages (CAR-M) are genetically engineered macrophages that express chimeric antigen receptors (CARs), similar to CAR-T cells used in cancer therapy. CAR-Ms are designed to target specific antigens on the surface of cancer cells. Upon binding to their target, CAR-Ms can engulf and digest cancer cells, as well as initiate a broader immune response by presenting tumor antigens to other immune cells, such as T cells [80]. Unlike CAR-T cells, CAR-Ms can directly phagocytose (engulf and digest) cancer cells. This gives them a unique advantage in directly eliminating tumor cells [83]. CAR-Ms can process and present tumor antigens to T cells, potentially triggering a broader and more sustained immune response against the tumor [83]. CAR-Ms can modulate the tumor microenvironment, converting it from an immunosuppressive state to one that supports anti-tumor immunity. This is particularly important in solid tumors, which often have a hostile microenvironment that impedes the effectiveness of traditional immunotherapies [80].

One of the challenges in developing CAR-M therapies is ensuring that the engineered macrophages can be effectively delivered to the tumor site, and that they persist long enough to exert their therapeutic effects [83,84]. Like all cell-based therapies, there is a concern about potential off-target effects or unintended immune reactions. Ensuring the safety of CAR-M therapies will be crucial as they move toward clinical trials [83,84]. The production of CAR-Ms involves complex genetic engineering and cell culture processes. Optimizing the manufacturing process to produce CAR-Ms at scale and at a reasonable cost is an ongoing challenge [80,83]. CAR-M therapies are currently in the preclinical stages and early-phase clinical trials. Early results have shown promise in terms of efficacy against solid tumors, which are traditionally more resistant to other forms of immunotherapy, such as CAR-T cells [83]. There is growing interest in combining CAR-M therapy with other treatments, such as immune checkpoint inhibitors or conventional chemotherapy, to enhance overall treatment efficacy. These combinations could potentially overcome resistance mechanisms and improve patient outcomes [84]. Macrophages, particularly in the form of CAR-M therapies, represent a promising frontier in cancer immunotherapy and other clinical applications. Their ability to directly target and eliminate tumor cells, reprogram the tumor microenvironment, and stimulate a broad immune response gives them unique advantages over existing therapies. However, challenges related to delivery, safety, and manufacturing need to be addressed before CAR-Ms can become a standard part of clinical practice. As research progresses, CAR-M therapies could play an important role in the treatment of cancers and potentially other diseases where macrophages are key players [80,81,82,83,84].

### 2.11. Summary the Proposed Therapies

Extended literature: TIM-3 [85,86,87,88,89], TIGIT [90,91], small molecule inhibitors [19,23,92], PI3K inhibitors [93], NFkB inhibitors [47,94], CD47 Inhibitors [52,95], Neddylation Inhibitors [57,96]. A final Table 13 is presented to outline the strengths and weaknesses of each therapeutic strategy in comparison to the others.

## 3. Innovative Combination Therapies for Hematologic Malignancies: Enhancing Treatment Efficacy and Overcoming Resistance 

### 3.1. PI3K Inhibitors in Combination Therapies

The constitutively active PI3K/AKT/mTOR pathway significantly contributes to the growth and survival of malignant cells by promoting the activation of several pro-survival and proliferative genes [24]. Combining a proteasome-inhibiting peptide like bortezomib with a PI3K inhibitor like idelalisib may result in a synergistic anti-cancer impact [97]. PI3K inhibitors can impede the signaling pathway, regulating cancer cell growth and proliferation, while proteasome inhibitors target the degradation of key proteins vital for cancer cell survival [98].

Pairing an immunological checkpoint inhibitor, like TIM-3 or TIGIT, with a PI3K inhibitor could boost the activation of the immune response against tumor cells and suppress their proliferation [61]. PI3K inhibitors may mitigate the immunosuppressive tumor microenvironment, while immunological checkpoint inhibitors may enhance T lymphocyte activation against tumor cells. Another discussed possibility is a regimen including a PI3K Inhibitor with a TIM-3 Inhibitor, where the PI3K inhibitor opposes cell signaling crucial for tumor cell survival, while the TIM-3 inhibitor promotes increased tumor cell apoptosis and enhanced activation of T lymphocytes against tumor cells [99]. 

Recent research has highlighted combining PI3K inhibitors with CAR-T cell therapy [100]. Idealisib, a potential inhibitor of PI3K, is also involved in the proliferation and function of T cells, both in vitro and in vivo. This small molecule inhibitor is also known to enhance the quality and function of T cells. Considering these results, it is feasible to utilize a PI3K inhibitor, which can regulate cancer cell proliferation, alongside CAR-T therapy that modifies patient T cells to target and eliminate tumor cells with enhanced efficacy. Furthermore, additional assistance from activated neutrophils could potentially enhance the efficacy of immunotherapy through synergistic neutrophil-mediated tumor elimination [100]. 

### 3.2. Immunological Checkpoint Inhibitors in Combination Therapies

Utilizing immune checkpoint inhibitors alongside proteasome inhibitors has been suggested in the literature. For instance, pairing an immunological checkpoint inhibitor, like PD-1 or CTLA-4 inhibitor, with a proteasome inhibitor such as bortezomib, holds promise for boosting the immune response against tumor cells and augmenting tumor cell apoptosis through the regulation of apoptosis-controlling protein expression [101]. 

Another potential approach to avoid resistance or functional dysregulation includes the combination of immune checkpoint inhibitors with signaling pathway inhibitors. Published data reveal that ibrutinib developed resistance in tumor-bearing mice, however, the combination of ibrutinib with an anti PD-L1 antibody inhibited cancer growth [102]. Similarly, an investigation employing the Eµ-TCL1 adoptive transfer mice model of CLL found that ibrutinib, in conjunction with antibodies that block the PD-1/PD-L1 axis, enhanced the activity of CD8 T cell effectors and the regulation of lymphocyte proliferation in vivo. This study indicated that ibrutinib’s potent immunomodulatory effects, combined with immune checkpoint inhibition, represent a promising treatment strategy for CLL [103] (Figure 4).

Additional evidence from research on chronic myeloid leukemia (CML) indicates that dasatinib treatment leads to Treg inhibition, a decreased abundance of myeloid-derived suppressor cells (MDSCs), and augmented NK cell differentiation and Granzyme B-expressing CD4+ and CD8+ memory T cells. This suggests Src family kinases (SFKs) play a role in immunosuppression during cancer progression. Based on these preclinical findings, anti-SFK tyrosine kinase inhibitors (TKIs), in combination with anti-PD-1 or PD-L1 ICIs, are currently being investigated in clinical trials for CML patients. Furthermore, dasatinib treatment boosted CD8+ T cell infiltration, decreased intra-tumoral Treg accumulation, and slowed tumor growth in syngeneic animal models of melanoma, sarcoma, colon, and breast cancer [104].

### 3.3. NF-κB Inhibitors in Combination Therapies

Combining NF-κB inhibitors with various therapeutic approaches offers a promising strategy for enhancing cancer treatment efficacy by targeting multiple pathways involved in tumor growth and survival. For example, combining an NF-κB inhibitor with TIGIT inhibitors can diminish tumor cell proliferation by regulating signaling pathways and inhibiting the immunosuppressive effects of regulatory T cells, resulting in heightened activation of the immune response against tumor cells [105]. 

Utilizing an NF-κB inhibitor to impede signaling pathways promoting cancer cell survival, in conjunction with monoclonal antibody therapy targeting tumor cell receptors, is also considered an efficient mechanism for tumor eradication. Sum et al. recently designed a bispecific anti-CD40 agonistic antibody that promotes T-cell priming via a dual mode of action by augmenting antigen delivery to macrophages, eventually resulting in their activation and tumor eradication [106]. Overall, with additional support from activated macrophages, this approach may amplify the effectiveness of anti-tumor therapy through enhanced macrophage-mediated tumor cell destruction. Tumor cell apoptosis may be enhanced, and an immune response against the tumor may be triggered by using a neddylation inhibitor. This can be achieved by suppressing the growth of malignant cells by triggering G2 cell cycle arrest and inducing DNA damage [107]. Furthermore, this strategy could boost tumor cell apoptosis and trigger an immune response against the tumor by impeding cancer cell growth and replication. 

Combining NF-κB inhibitors with proteasome inhibitors, such as bortezomib, impedes signaling pathways supporting cancer cell survival while targeting cancer cell surface receptors. This regimen may yield a synergistic anti-tumor impact [108]. Similarly, combining NF-κB inhibitors with hyperthermia therapy and gene therapy increases treatment sensitivity and induces apoptosis, providing a multi-faceted approach to limiting tumor growth [109]. 

NF-κB inhibitors also play a significant role in targeting angiogenesis. They regulate the transcription of several genes involved in vascular differentiation, proliferation, apoptosis, and tumorigenesis. By modulating angiogenic factor expression levels, particularly VEGF, NF-κB controls the development of several carcinomas. Blocking NF-κB signals greatly reduces VEGF, IL-8, and MMP-9-induced tumor angiogenesis, both in vitro and in vivo. Combining an NF-κB inhibitor, which blocks signaling pathways promoting tumor cell survival, with hyperthermic therapy, gene therapy, and biological therapies focused on angiogenesis inhibition, could prove a potential anti-angiogenesis therapy for certain malignancies [41]. 

Apart from NF-κB signaling inhibitor, the neddylation inhibitor MLN4924 can also inhibit cell proliferation by interfering with cell cycle checkpoint regulators, p21, p27, and phospho-histone H3 [110]. Moreover, recent research reveals that MLN4924, in combination with ibrutinib (a tyrosine kinase inhibitor), demonstrated safety and promising efficacy towards NHL and CLL [110]. Overall, using a neddylation inhibitor, which prevents the degradation of proteins regulating tumor cell growth, along with an immunotherapy, could potentially improve treatment efficacy and therapeutic outcomes [111].

Combining NF-κB inhibitors with TIGIT inhibitors, monoclonal antibodies, proteasome inhibitors, hyperthermia therapy, gene therapy, and neddylation inhibitors, as well as targeting angiogenesis, presents multiple synergistic strategies to enhance cancer treatment efficacy and overcome resistance mechanisms.

### 3.4. Neddylation Inhibitors in Combination Therapies

Using neddylation inhibitors in combination therapies presents a promising approach to enhance the efficacy of cancer treatments by targeting multiple pathways crucial for tumor cell survival and proliferation. Neddylation inhibitors, which block the degradation of protein crucial for tumor cell survival, can be combined with various other therapeutic agents to improve treatment outcomes [Figure 5]. 

Combining neddylation inhibitors with tumorigenesis inhibitors could enhance tumor cell apoptosis and restrain tumor growth and metastasis. Such combinations are currently in the preliminary stages and necessitate further investigation to evaluate their efficacy and safety, particularly in patients with hematologic malignancies. These combinations might unveil novel avenues for treating these diseases [112]. 

Another innovative approach involves the combination of neddylation inhibitors with antigen complex therapy and nuclease inhibitors. This combination therapy would block the degradation of cancer survival proteins, involve tumor cell antigen presentation to elicit an immune response, and hinder DNA replication in neoplastic cells. Consequently, tumor cell apoptosis is increased, and an immune response against the tumor can be mounted. While these complex therapeutic combinations represent novel approaches in the treatment of hematologic malignancies, their efficacy and safety require further study [113]. 

Furthermore, combining neddylation inhibitors with molecularly targeted therapy for the tumor microenvironment and hormonal therapy offers unique benefits. This regimen utilizes molecularly targeted therapy to interact with the tumor microenvironment, reducing metabolic stress in surrounding cells. When supplemented with hormonal therapy, tumor cell growth is impeded, and treatment efficacy is enhanced [96]. 

The combination of neddylation inhibitors with tumorigenesis inhibitors, antigen complex therapy, nuclease inhibitors, molecularly targeted therapy, and/or hormonal therapy represents an innovative strategy to enhance cancer treatment efficacy. These combinations hold potential for improving therapeutic outcomes by targeting multiple pathways involved in tumor cell survival and proliferation (Figure 5).

### 3.5. Summary of Combination Therapies for Hematologic Malignancies

Table 14 provides a summary of combination therapies used in the treatment of hematologic malignancies.

## 4. Other Innovative Combination Therapies for Hematologic Malignancies 

Combining CAR-T cell therapy with immune checkpoint inhibitors, such as pembrolizumab (anti-PD-1), has been shown to enhance the persistence and function of CAR-T cells. This approach addresses challenges like T cell exhaustion and improves the overall anti-tumor efficacy. Patients with hematological malignancies benefit from a more robust immune response against cancer cells when these therapies are combined [114,115].

Radioimmunotherapy, which integrates radiotherapy with immunotherapy by using radioactive substances attached to antibodies, specifically targets cancer cells. This combination not only destroys tumor cells but also enhances the immunogenicity of the tumor microenvironment, making it more susceptible to immune attack. The addition of CAR-T cells and immune checkpoint inhibitors further amplifies this effect, leading to improved tumor control [116].

Interferon-based treatments, particularly interferon-alpha, boost the immune system’s response to cancer by stimulating immune cells, increasing antigen presentation, and inhibiting tumor cell proliferation. When combined with CAR-T cells and checkpoint inhibitors, interferons significantly enhance the anti-tumor immune response, improving treatment outcomes [115].

The CD47 “Don’t Eat Me” blockade enhances the phagocytosis of cancer cells through macrophages, overcoming a key immune evasion mechanism used by tumors. Combining CD47 inhibitors with CAR-T cells and checkpoint inhibitors has shown promising results in preclinical studies, enhancing both innate and adaptive immune responses against tumors. This multifaceted approach helps with the effective elimination of cancer cells [117,118].

Combining immune checkpoint inhibitors with signaling pathway inhibitors, such as ibrutinib, addresses resistance mechanisms in hematologic malignancies. For example, ibrutinib combined with PD-1/PD-L1 blockade enhances the activity of CD8 T cells and regulates lymphocyte proliferation, offering a potent treatment strategy for CLL and other cancers [115].

Using metabolic inhibitors and epigenetic modifiers in conjunction with immunotherapies can improve tumor antigen expression and disrupt cancer cell metabolism. This approach enhances immune recognition and response, making tumors more susceptible to immune-mediated destruction [118].

Combining angiogenesis inhibitors with immune checkpoint inhibitors reduces tumor vascularization and enhances immune cell infiltration. Additionally, TLR agonists can activate both innate and adaptive immune responses, further improving the efficacy of immunotherapies. These innovative combination therapies provide a comprehensive approach to tackling hematologic malignancies, aiming to improve patient outcomes through enhanced therapeutic efficacy and overcoming resistance mechanisms [115,118].

### Summary Table

For other potential combination therapies for hematologic malignancies, please refer to Table 15 below.

## 5. Potentially Toxic Therapies

Three of the most potentially toxic therapies in cancer treatment are radioimmunotherapy, gene therapy, and hyperthermic therapy. While these treatments offer significant therapeutic benefits, they carry inherent risks that necessitate vigilant monitoring to mitigate potential adverse effects. Radioimmunotherapy can cause radiation-induced damage to surround healthy tissues, leading to side effects such as fatigue, nausea, and an increased risk of secondary cancers [119]. Gene therapy carries risks of unintended consequences such as the development of new malignancies or immune reactions, and the vectors used may provoke immune responses or cause other side effects [120]. Hyperthermic therapy can lead to potential risks including damage to normal tissues and organs, dehydration, and heat stroke, with side effects such as pain, swelling, and burns at the treatment site [121].

In addition to these therapies, other potentially toxic or combined therapies include stem cell transplantation, combination chemotherapy, and targeted therapy with immunotherapy combinations. Stem cell transplantation poses toxicity risks including infections, graft-versus-host disease, and organ damage, requiring rigorous patient monitoring and supportive care [122]. Combination chemotherapy can increase toxicity with severe side effects including bone marrow suppression, gastrointestinal disturbances, and cardiotoxicity, necessitating dose adjustments and supportive therapies. Combining targeted therapies with immunotherapies can exacerbate immune-related adverse effects such as severe skin reactions, colitis, hepatitis, and pneumonitis, requiring continuous monitoring and prompt management of side effects [123].

In conclusion, while these innovative therapies offer promising approaches to cancer treatment, they present significant potential toxicities (Table 16). Ensuring patient safety requires meticulous planning, precise delivery of therapy, and continuous monitoring to manage and mitigate these risks effectively. 

## 6. Discussion

The advent of combination therapies represents a paradigm shift in the management of hematologic malignancies, offering new avenues to address treatment resistance and improve patient outcomes. By strategically integrating targeted agents, immunotherapies, and conventional treatments, approaches capitalize on synergistic interactions to enhance efficacy and overcome tumor evasion mechanisms. Combination therapies have significantly advanced the management of hematologic malignancies by integrating targeted agents, immunotherapies, and conventional treatments to enhance patient outcomes and combat treatment resistance [6,124]. For example, the combination of histone deacetylase (HDAC) inhibitors and Enhancer of Zeste Homologue 2 (EZH2) inhibitors has shown promise in hematological malignancies [124]. Additionally, the integration of therapies such as chemotherapy, stem cell transplantation, radiotherapy, and immunotherapy has notably improved the prognosis of hematologic malignancies [6]. 

One of the challenges in optimizing combination therapies is addressing therapy resistance mechanisms. For instance, BCL2 inhibition by venetoclax has emerged as a new treatment approach in various hematological malignancies, underscoring the importance of understanding and overcoming resistance mechanisms [125]. Moreover, the use of monoclonal antibodies against Eph family receptors has gained attention as an effective therapeutic strategy in hematological tumors, emphasizing the significance of targeted therapies in addressing resistance and improving outcomes [126]. The exploration of epigenetic drugs and their combinations in myeloid malignancies highlights the trend towards combination therapies to enhance drug synergy and overcome resistance [127]. 

Combining a neddylation inhibitor with complex antigen therapy and a nuclease inhibitor, bolstered by macrophages: employing a neddylation inhibitor to prevent the degradation of proteins regulating tumor cell growth, along with complex antigen therapy presenting tumor antigens to the immune system and a nuclease inhibitor hindering DNA replication in cancer cells, with added support from activated macrophages, might augment the efficacy of anti-tumor therapy through heightened macrophage-mediated tumor cell elimination [107].

Innovative combination therapies for hematologic malignancies present an opportunity to significantly improve treatment outcomes by targeting cancer cells from multiple angles. One such proposed combination includes the use of PI3K inhibitors, autophagy inhibitors, and immune checkpoint inhibitors. Idelalisib, a PI3K inhibitor, disrupts a crucial pathway for cancer cell growth and survival. Chloroquine, an autophagy inhibitor, blocks the process cancer cells use to survive under metabolic stress. Pembrolizumab, a PD-1 inhibitor, enhances the immune response by preventing the PD-1 receptor on T cells from being activated, thus allowing the immune system to more effectively target cancer cells. This combination aims to increase cancer cell death by targeting their metabolic pathways, survival mechanisms, and immune evasion strategies. Currently, no clinical trials have tested this specific combination, making it a novel approach in the treatment of hematologic cancers.

Another promising combination includes NF-κB inhibitors, neddylation inhibitors, and immune checkpoint inhibitors. Bay 11-7082, an NF-κB inhibitor, reduces cancer cell survival and proliferation by blocking the NF-κB pathway. Pevonedistat, a neddylation inhibitor, disrupts protein degradation and promotes apoptosis. Nivolumab, a PD-1 inhibitor, enhances the immune response by blocking the PD-1 receptor on T cells. This combination targets multiple pathways crucial for cancer cell survival and proliferation, providing a multifaceted approach to treating cancer and potentially overcoming resistance. To date, no clinical trials have tested this specific combination.

Lastly, the combination of CD47 blockade, MET inhibitors, and anti-CTLA-4 therapy holds promise. Hu5F9-G4, a CD47 inhibitor, enhances the phagocytosis of cancer cells through macrophages. Tepotinib, a MET inhibitor, targets the MET pathway involved in tumor growth and metastasis. Ipilimumab, a CTLA-4 inhibitor, increases T cell activity against cancer cells by blocking CTLA-4. This combination aims to engage both the innate and adaptive immune systems, leading to enhanced phagocytosis, reduced tumor growth and metastasis, and a stronger immune response. This combination is theoretically robust but has not been studied in clinical trials.

These proposed combinations leverage multiple therapeutic strategies to improve efficacy and overcome resistance mechanisms in hematologic malignancies. While individual components have been tested in various contexts, these specific combinations remain unexplored, presenting opportunities for innovative research.

Combination chemotherapy regimens pose a notable challenge due to the potential for increased toxicity resulting from the additive or synergistic effects of individual agents, which may lead to heightened adverse events [128]. The toxicity associated with these regimens can be substantial, with reports of up to 50–70% of grade 3–4 neutropenia [129]. To address these challenges, careful patient selection, dose optimization, and monitoring strategies are crucial to mitigate risks and ensure tolerability while maximizing therapeutic benefit. Moreover, the identification of predictive biomarkers and the development of personalized treatment algorithms are essential to tailor combination therapies to individual patient profiles, optimizing efficacy and minimizing toxicity [130]. The development of combination regimens, although desirable for enhanced efficacy, poses unique challenges, particularly in managing toxicities. Rigorously conducted comparative studies or network meta-analyses of patient-level data are necessary to fully understand the comparative benefits and harms of different combination chemotherapy regimens [131]. Researchers aim to combine drugs that are effective as single agents and exhibit synergistic behavior when combined, avoiding combinations of drugs that cause similar toxic effects or have the same patterns of resistance [132]. While combination regimens have shown superiority over sequential single regimens, it is crucial to balance the increased efficacy with the potential for greater toxicity. Quadruplet combinations in certain contexts have been associated with greater toxicity without additional therapeutic benefits [133]. Therefore, a comprehensive understanding of the toxicity profiles of different regimens is essential for informing optimal supportive care and future clinical trial design [134]. 

The optimization of combination chemotherapy regimens requires a delicate balance between maximizing therapeutic efficacy and minimizing toxicity. Careful consideration of patient characteristics, dose adjustments, monitoring strategies, and the identification of predictive biomarkers are vital components in tailoring combination therapies to individual patients, ensuring the best possible outcomes while managing potential toxicities. Our discussion underscores the transformative potential of integrating diverse therapeutic strategies, encompassing immunotherapies, molecular therapies, and interventions targeting the tumor microenvironment. Incorporating CD47 receptor inhibitors into these therapeutic regimens may engender a synergistic anti-tumor effect, bolstering the immune system’s capacity to eradicate cancer cells [135].

The interplay between local nanomechanical properties and the red blood cell (RBC) cytoskeleton is crucial for understanding the biomechanical behavior of cells and how they can be affected by therapeutic interventions, particularly in the context of hematologic diseases and treatments like CAR-T therapy [136,137]. Local nanomechanical properties refer to the mechanical characteristics at the nanoscale, such as stiffness, elasticity, and viscosity, which can be measured using techniques like atomic force microscopy (AFM) and optical tweezers. These properties are essential for determining how cells respond to external forces, interact with their environment, and maintain their structural integrity [136,137]. Monitoring these properties can provide insights into the disease state and the effectiveness of treatment. The RBC cytoskeleton, composed mainly of spectrin, actin, and ankyrin, among other proteins, is a complex network of proteins that provides structural support, maintains the biconcave shape of RBCs, and allows for their deformability, which is crucial for their function in microcirculation [138]. Disruptions in the cytoskeleton, as seen in various hematologic disorders (e.g., hereditary spherocytosis, elliptocytosis, and sickle cell disease), lead to altered mechanical properties such as increased stiffness or reduced deformability. These changes can be detected using AFM, providing a non-invasive way to assess the state of the cytoskeleton [136,137].

CAR-T therapy, a form of immunotherapy where T cells are engineered to target and destroy cancer cells, has revolutionized the treatment of certain hematologic malignancies like leukemia and lymphoma [138]. Changes in the nanomechanical properties of RBCs and other cells in the blood could serve as early biomarkers for the onset and proliferation of hematolytic diseases or for monitoring the effects of therapies like CAR-T [139]. If these nanomechanical changes can be quantitatively linked to specific stages of disease or responses to therapy, they could be used to personalize treatment plans, improving outcomes by adjusting therapies before significant side effects or disease progression occurs [136,137].

Nevertheless, as we embrace these novel discoveries, we also encounter fresh challenges, including the need to monitor and mitigate adverse effects while customizing treatments to suit individual patient profiles. Further clinical investigations are imperative to ascertain the efficacy and safety profiles of these innovative therapeutic combinations, thereby delineating optimal treatment modalities for distinct cancer subtypes. 

The integration of real-world evidence and patient-centered approaches are also critical aspects of advancing combination therapies. Real-world data can provide insights into how therapies perform outside the controlled environment of clinical trials, reflecting long-term effectiveness and safety across diverse patient populations [140]. Furthermore, engaging patients in the treatment planning process improves adherence to therapy and satisfaction with outcomes, as patient preferences and quality of life measures are increasingly considered in clinical trial designs [141].

In conclusion, the advent of combination therapies represents a significant advancement in the field of hematologic malignancies, providing a comprehensive approach to address treatment resistance and improve patient prognosis. By strategically integrating various treatment modalities, researchers and clinicians aim to capitalize on synergistic interactions, overcome resistance mechanisms, and ultimately enhance the efficacy of therapeutic interventions.

## 7. Conclusions

In conclusion, combination therapies hold tremendous promise in the treatment of hematologic malignancies, offering novel strategies to overcome resistance and improve outcomes for patients. By integrating targeted agents, immunotherapies, and conventional treatments, these approaches capitalize on synergistic interactions to enhance efficacy and combat tumor evasion mechanisms. For instance, combining PI3K inhibitors, autophagy inhibitors, and immune checkpoint inhibitors presents a multifaceted attack on cancer cells, disrupting their survival pathways, metabolic processes, and immune evasion strategies. Similarly, the combination of NF-κB inhibitors, neddylation inhibitors, and immune checkpoint inhibitors targets multiple survival pathways, potentially overcoming resistance mechanisms. Furthermore, the integration of CD47 blockade, MET inhibitors, and anti-CTLA-4 therapy engages both the innate and adaptive immune systems, enhancing phagocytosis, reducing tumor growth, and strengthening immune responses. 

While combination therapies show significant potential, challenges such as toxicity and therapy resistance remain. Careful patient selection, dose optimization, and monitoring strategies are essential to mitigate these risks.

Moreover, incorporating real-world evidence and patient-centered approaches into combination therapy development is essential. Real-world data provide insights into the long-term effectiveness and safety of therapies across diverse patient populations [140], while patients engaging in treatment planning improves adherence and satisfaction with outcomes [141].

By addressing these challenges and leveraging interdisciplinary collaboration, we can unlock the full potential of combination therapies, ushering in a new era of precision medicine in hematologic oncology. Further clinical investigations are imperative to validate the efficacy and safety profiles of these innovative combinations, delineating optimal treatment modalities for distinct cancer subtypes.

The transformative potential of these approaches lies in their ability to integrate diverse therapeutic strategies, encompassing immunotherapies, molecular therapies, and interventions targeting the tumor microenvironment, ultimately enhancing the efficacy of therapeutic interventions in hematologic malignancies.

The future of combinational therapies in hematological malignancies is promising, with advances in personalized medicine, immunotherapy, and targeted drug development leading the way. By leveraging cutting-edge technologies and a deeper understanding of cancer biology, these therapies are expected to offer more effective, less toxic, and highly personalized treatment options for patients with hematologic cancers. Combinational therapies in hematological malignancies have become a cornerstone of treatment strategies, offering improved efficacy, reduced resistance, and better patient outcomes. The future directions for combinational therapies in these diseases are likely to be shaped by advances in personalized medicine, novel drug development, and a deeper understanding of the molecular and immune landscapes of these cancers. Here in Table 17 we present some of the future directions.

## Figures and Tables

**Table 1:** TIM-3 inhibitors and antibodies in clinical development for solid tumors and hematologic malignancies: mechanisms of action, targets, and clinical applications.

Drug	Mechanism of Action/Target/Type of Molecule	Type of Malignancy	Approval Status	Combination Therapies	Adverse Effects
MBG453	TIM-3 inhibitor/Antibody	Lymphomas, advanced solid tumors	Clinical Trials II	Investigated with other immune checkpoint inhibitors	Fatigue, nausea
Sym023	TIM-3 inhibitor/Monoclonal antibody	Advanced solid tumors, lymphomas	Clinical Trials I/II	Research ongoing for combinations	Fatigue, infusion reactions
LY3321367	TIM-3 inhibitor/Antibody	Solid tumors, hematologic malignancies	Clinical Trials I/II	Being studied with other therapies	Under investigation
LY3415244	Anti-TIM-3 antibody	Solid tumors and hematologic malignancies	Clinical Trials I/II	Combination immunotherapy	Under investigation
TSR-022	Anti-TIM-3 antibody	Solid tumors and lymphomas	Clinical Trials I/II	Being tested with other checkpoint inhibitors	Under investigation
Lomvastomig: RO7121661	TIM-3 inhibitor/Bispecific antibody	Advanced solid tumors and lymphomas	Clinical Trials I/II	Research ongoing for combinations	Under investigation
TSR-042	Anti-PD-1 and TIM-3 antibody	Various cancers	Clinical Trials II	Being studied with other immunotherapies	Fatigue, immune-related adverse events

**Table 2:** TIGIT inhibitors and related agents in clinical development for hematological malignancies. Abbreviations: Acute Myeloid Leukemia (AML), Chronic Lymphocytic Leukemia (CLL), Hodgkin Lymphoma (HL), Non-Hodgkin lymphoma (NHL), Multiple Myeloma (MM).

Agent Name	Mechanism of Action	Hematological Malignancies	Clinical Development Phase
Tiragolumab	Anti-TIGIT monoclonal antibody	NHL, MM	Phase II
Vibostolimab	Anti-TIGIT monoclonal antibody	HL	Phase II
Ociperlimab	Anti-TIGIT monoclonal antibody	AML	Phase I/II
AGEN1777	Bispecific antibody targeting TIGIT and PD-1	MM, NHL	Phase I/II
COM902	Anti-TIGIT monoclonal antibody	MM	Phase I
AB154	Anti-TIGIT monoclonal antibody	CLL	Phase I

**Table 3:** Small molecule inhibitors and their targets in hematologic malignancies. Abbreviations: Acute Myeloid Leukemia (AML), Chronic Lymphocytic Leukemia (CLL), Diffuse Large B-Cell Lymphoma (DLBCL), Follicular Lymphoma (FL), Mantle Cell Lymphoma (MCL), Marginal Zone Lymphoma (MZL), Small Lymphocytic Lymphoma (SLL).

Name of Molecule	Mechanism of Action	Type of Hematologic Malignancy	Approval Status	Clinical Trial Phase	Adverse Effects
Idelalisib (Zydelig)	Inhibits PI3Kδ, reducing cell proliferation and survival signals	CLL, FL, SLL	FDA Approved	N/A	Diarrhea, liver toxicity
Copanlisib (Aliqopa)	Inhibits PI3Kα and PI3Kδ, affecting cell growth and survival	FL	FDA Approved	N/A	Hyperglycemia, hypertension
Duvelisib (Copiktra)	Inhibits PI3Kδ and PI3Kγ, reducing cytokine synthesis and promoting apoptosis	CLL, SLL	FDA Approved	N/A	Diarrhea, colitis
TGR-1202 (Umbralisib)	Inhibits PI3Kδ and casein kinase-1ε (CK1ε), affecting cell adhesion and migration	CLL, MZL, FL	FDA Approved	N/A	Diarrhea, nausea
Zandelisib (ME-401)	Inhibits PI3Kδ, affecting cell proliferation and survival signals	FL, CLL, SLL, MZL, DLBCL	Clinical Trials	II/III	Diarrhea, liver toxicity
Linperlisib	Inhibits PI3Kδ, reducing cell proliferation and survival signals	FL	Clinical Trials	I/II	Fatigue, nausea
TQB3525	Inhibits PI3Kα and PI3Kδ, affecting cell growth and survival	CLL, SLL	Clinical Trials	I/II	Under investigation
Acalisib	Inhibits PI3Kδ, reducing cell proliferation and survival signals	FL, DLBCL, MZL, MCL	Clinical Trials	I	Under investigation
SHC014748M	Inhibits PI3Kδ, reducing cell proliferation and survival signals	CLL	Preclinical	N/A	Under investigation
Venetoclax	Inhibits BCL-2, promoting apoptosis in cancer cells	CLL, AML	FDA Approved	N/A	Neutropenia, infections
Selinexor	Inhibits XPO1, blocking nuclear export and leading to apoptosis	MM, DLBCL	FDA Approved	N/A	Nausea, fatigue
Bortezomib	Inhibits proteasome activity, leading to the accumulation of pro-apoptotic proteins and triggering apoptosis	MM	FDA Approved	N/A	Peripheral neuropathy, fatigue
Melphalan	Binds at the N7 position of guanine, inducing inter-strand cross-links in DNA	MM	FDA Approved	N/A	Bone marrow suppression
P5091	Inhibits USP7, blocking HDM2 and p21 signaling pathways	MM	Clinical Trials	I/II	Well tolerated in studies

**Table 4:** Overview of PI3K inhibitors in the treatment of hematologic malignancies: mechanisms of action and clinical applications. Abbreviations: Chronic Lymphocytic Leukemia (CLL), Diffuse Large B-Cell Lymphoma (DLBCL), Follicular Lymphoma (FL), Marginal Zone Lymphoma (MZL), Small Lymphocytic Lymphoma (SLL).

Name of Molecule	Target	Type of Malignancy	Approval Status	Mechanism of Action	Combination Therapies	Adverse Effects
Idelalisib (Zydelig)	Inhibitor of PI3K-Delta	CLL, FL, SLL	FDA Approved	Induces caspase-dependent apoptosis	Being studied with anti-CD20 antibodies	Diarrhea, colitis, liver toxicity
Copanlisib (Aliqopa)	Inhibitor of PI3K-alpha and PI3K delta	FL	FDA Approved	Inhibits PI3K signaling, affecting cell proliferation and survival	Used with rituximab	Hyperglycemia, hypertension
Duvelisib (Copiktra)	Inhibitor of PI3K-delta and PI3K-gamma	CLL and SLL	FDA Approved	Reduces cytokine synthesis, direct cytotoxicity to leukemic cells	Investigated with BTK inhibitors	Diarrhea, colitis, pneumonitis
TGR-1202 (Umbralisib)	Dual PI3Kδ and CK1ε inhibitor	CLL, MZL, FL	FDA Approved	Inhibits PI3Kδ and CK1ε, reduces tumor cell adhesion and migration	Combined with BTK inhibitors	Diarrhea, nausea, fatigue
Zandelisib (ME-401)	Inhibitor of PI3K-Delta	FL, CLL, SLL, MZL, DLBCL	Phase II/III Clinical	Inhibits PI3K signaling, affecting cell proliferation and survival	Being tested with rituximab	Diarrhea, liver toxicity
Linperlisib	Inhibitor of PI3K-Delta	FL	Phase I/II Clinical	Inhibits PI3K signaling pathways	Combined with other chemotherapies	Fatigue, nausea
TQB3525	Inhibitor of PI3K-alpha and PI3K delta	CLL, SLL	Phase I/II Clinical	Targets PI3K signaling, affects cell survival	Research ongoing for combinations	Under investigation
Acalisib	Inhibitor of PI3K-Delta	FL, DLBCL, MZL, MCL	Phase I Clinical	Inhibits PI3Kδ, impacting cell survival	Studied with chemotherapy agents	Under investigation
SHC014748M	Inhibitor of PI3K-Delta	CLL	Preclinical	Targets PI3K signaling pathways	Potential for combination therapy	Under investigation

**Table 5:** Inhibitors targeting the NF-κB pathway in hematologic malignancies. Abbreviations: Acute Lymphoblastic Leukemia (ALL), Lymphomas (L), Multiple Myeloma (MM), Various Leukemias (VL).

Name of Molecule	Mechanism of Action	Type of Hematologic Malignancy	Type of Molecule	Approval Status	Combination Therapies	Adverse Effects
Curcumin	Inhibits NF-κB activation by suppressing various upstream signaling pathways	MM	Diarylheptanoid (curcuminoids group)	Preclinical	Research ongoing for combinations	Generally well tolerated
Bay 11-7082	Inhibits NF-κB activation by targeting the IκB kinase complex, preventing phosphorylation of IκBα	MM, L	IκB Kinase (IKK) inhibitor	Preclinical	Investigated with other inhibitors	Under investigation
Parthenolide	Inhibits NF-κB activation by targeting the IκB kinase complex, preventing phosphorylation of IκBα	ALL, L	Germacranolide	Preclinical	Investigated with other NF-κB inhibitors	Cytotoxicity at high doses
IKK Inhibitor MLN120B	Targets IKK complex, preventing phosphorylation of IκBα	VL and L	Small molecule inhibitor	Preclinical	Investigated with chemotherapies	Under investigation
Resveratrol	Inhibits NF-κB activation by suppressing phosphorylation and degradation of IκBα, preventing NF-κB translocation	VL and L	Polyphenolic phytoalexin (Stilbene class)	Clinical Trials I/II	Combined with chemotherapies	Mild gastrointestinal symptoms

**Table 6:** CD47 Inhibitors and their targets in hematologic malignancies.

Name of Molecule	Target	Type of Malignancy	Type of Molecule	Approval Status	Combination Therapies	Adverse Effects
Hu5F9-G4	Selectively binds to CD47 expressed on tumor cells and blocks the interaction with SIRPa	AML, MM, LBCL, and some solid tumors	Peptide (monoclonal antibody)	Clinical Trials I/II	Investigated with other chemotherapies	Anemia, fatigue
SIRPαFc (TTI-621)	Binds to CD47 on tumor cells, preventing inhibitory signals to macrophages, and engages FcγR to enhance phagocytosis	Relapsed/refractory hematologic malignancies and solid tumors	Peptide	Clinical Trials I/II	Combined with other immune checkpoint inhibitors	Thrombocytopenia, anemia
CC-90002	Anti-CD47 antibody that inhibits CD47-SIRPα interaction, enabling macrophage-mediated killing of tumor cells	Relapsed/refractory hematologic malignancies and solid tumors	Peptide (antibody)	Clinical Trials I/II	Investigated with other mAbs	Cytokine release syndrome
ALX148	Enhances macrophage phagocytosis of tumor cells and inhibits binding of wild-type SIRPα	Non-Hodgkin Lymphoma and solid tumors	Peptide (antibody)	Clinical Trials I/II	Combined with rituximab, pembrolizumab	Infusion reactions, anemia

**Table 7:** Neddylation inhibitors and their applications in hematologic malignancies. Abbreviations: Acute Myeloid Leukemia (AML), Multiple Myeloma (MM), Myelodysplastic Syndromes (MDS).

Name of Molecule	Mechanism of Action	Type of Hematologic Malignancy	Type of Molecule	Approval Status	Combination Therapies	Adverse Effects
Pevonedistat (MLN4924)	Inhibits NEDD8-activating enzyme (NAE), disrupting neddylation, inducing apoptosis, senescence, and autophagy via p53 pathway activation	AML, MM, MDS	NEDD8-activating enzyme inhibitor	Clinical Trials II/III	Investigated with chemotherapies and immunotherapies	Nausea, fatigue, and hematologic toxicity
TAS4464	Selectively inhibits NAE, leading to cullin neddylation inhibition and accumulation of CRL substrates, inducing antiproliferative activity	AML, MM	NEDD8-activating enzyme inhibitor	Clinical Trials I/II	Investigated with molecular and hormonal therapies	Under investigation
MLN4924	Inhibits NAE, leading to the activation of the p53 signaling pathway and subsequent anti-leukemia effects	AML, MM	NEDD8-activating enzyme inhibitor	Clinical Trials II/III	Investigated with molecular, immunotherapy-based therapies	Nausea, fatigue, hematologic toxicity
TAS4464	Highly potent NAE inhibitor, inducing cullin neddylation inhibition and CRL substrate accumulation, leading to widespread antiproliferative activity	AML, MM	NEDD8-activating enzyme inhibitor	Clinical Trials I/II	Combined with molecular therapies, CD47 receptor blockade	Under investigation

**Table 8:** PD-1 inhibitors and their applications in hematologic malignancies. Abbreviations: Hodgkin Lymphoma (HL), Primary Mediastinal Large B-Cell Lymphoma (PMBCL).

Name of Inhibitor	Clinical Trial Phase	Mechanism of Action	Type of Hematologic Malignancy	FDA Status	Combination Therapies	Adverse Effects
Nivolumab (Opdivo)	III/IV	Inhibits PD-1, preventing binding with PD-L1/PD-L2 and restoring T-cell activity	HL	Approved	Chemotherapy, targeted therapy, other immunotherapies	Fatigue, rash, diarrhea, hepatitis
Pembrolizumab (Keytruda)	III/IV	Inhibits PD-1, preventing binding with PD-L1/PD-L2 and restoring T-cell activity	HL, PMBCL	Approved	Chemotherapy, targeted therapy, other immunotherapies	Fatigue, pruritus, rash, pneumonitis
Cemiplimab (Libtayo)	III/IV	Inhibits PD-1, preventing binding with PD-L1/PD-L2 and restoring T-cell activity	HL	Clinical Trials	Chemotherapy, targeted therapy	Fatigue, rash, musculoskeletal pain
Sintilimab	III/IV	Inhibits PD-1, preventing binding with PD-L1/PD-L2 and restoring T-cell activity	HL	Approved (China)	Chemotherapy, targeted therapy, other immunotherapies	Pyrexia, hypothyroidism, pneumonia
Toripalimab	III/IV	Inhibits PD-1, preventing binding with PD-L1/PD-L2 and restoring T-cell activity	HL	Approved (China)	Chemotherapy, targeted therapy, other immunotherapies	Fatigue, fever, hypothyroidism
Camrelizumab	III/IV	Inhibits PD-1, preventing binding with PD-L1/PD-L2 and restoring T-cell activity	HL	Approved (China)	Chemotherapy, targeted therapy, other immunotherapies	Rash, pruritus, arthralgia

**Table 9:** Therapeutic monoclonal antibodies in hematologic malignancies. Abbreviations: Anaplastic Large Cell Lymphoma (ALCL), B Cell Leukemia (BCL), B Cell Non-Hodgkin Lymphomas (BNHL), Chronic Lymphocytic Leukemia (CLL), Hodgkin Lymphoma (HL), Follicular Lymphoma (FC), Large B Cell Lymphoma (LBCL), Hematologic Malignancies (HM), Multiple Myeloma (MM), T Cell Lymphomas (TCL), T Cell Prolymphocytic Leukemia (T-PL).

Target	Applications	Mechanism of Action	Clinical Impact
Rituximab (Rituxan) CD20 antigen on B cells [64].	Rituximab is primarily used in the treatment of BNHL, including DLBCL and FL, as well as in CLL.	Rituximab binds to the CD20 antigen on B cells, leading to cell death through complement-dependent cytotoxicity (CDC), antibody-dependent cellular cytotoxicity (ADCC), and direct induction of apoptosis.	The introduction of Rituximab has significantly improved survival rates in B-cell malignancies. It is often used in combination with chemotherapy (e.g., the R-CHOP regimen) and as maintenance therapy to prevent relapse.
Daratumumab (Darzalex) CD38 antigen on plasma cells [65].	Daratumumab is widely used in the treatment of MM, both as a monotherapy and in combination with other agents like lenalidomide, bortezomib, and dexamethasone.	Daratumumab targets CD38, leading to cell death through CDC, ADCC, antibody-dependent cellular phagocytosis (ADCP), and apoptosis.	Daratumumab has transformed the treatment landscape for MM, offering significant improvements in progression-free survival and overall survival, particularly in relapsed and refractory settings.
Brentuximab Vedotin (Adcetris) CD30 antigen on Reed-Sternberg cells and some TCL [66].	Brentuximab Vedotin is used in the treatment of HL and certain types of TCL, including ALCL.	This antibody-drug conjugate (ADC) consists of a CD30-directed monoclonal antibody linked to the cytotoxic agent monomethyl auristatin E (MMAE). Upon binding to CD30, the conjugate is internalized, and MMAE is released, leading to cell cycle arrest and apoptosis.	Brentuximab Vedotin has shown high efficacy in relapsed and refractory HL and ALCL, providing an important treatment option, especially for patients who have failed conventional chemotherapy.
Inotuzumab Ozogamicin (Besponsa) CD22 antigen on B cells [67].	Inotuzumab Ozogamicin is used in the treatment of relapsed or refractory B-cell ALL.	This ADC targets CD22, delivering the cytotoxic antibiotic calicheamicin directly to the cancer cells, leading to DNA damage and cell death.	Inotuzumab Ozogamicin has improved outcomes in relapsed/refractory ALL, offering a targeted therapy option with high response rates in a difficult-to-treat patient population.
Elotuzumab (Empliciti) SLAMF7 (signaling lymphocytic activation molecule family member 7) on myeloma cells and natural killer (NK) cells [68].	Elotuzumab is used in combination with lenalidomide and dexamethasone for the treatment of MM, particularly in relapsed/refractory cases.	Elotuzumab enhances NK cell-mediated ADCC against SLAMF7-expressing myeloma cells, while also activating NK cells to attack the cancer cells.	Elotuzumab has been shown to improve progression-free survival in patients with MM, especially when used in combination therapy.
Alemtuzumab (Campath) CD52 antigen on B and T cells [69].	Alemtuzumab is used in the treatment of CLL and, in some cases, T-PL.	Alemtuzumab targets CD52, leading to cell death through CDC and ADCC.	Alemtuzumab has been effective in CLL, particularly in patients with 17p deletion who are typically resistant to other therapies. However, its use is limited due to significant immunosuppression and infection risks.

**Table 10:** CTLA-4 inhibitors and their applications in hematologic malignancies. Abbreviations: Various Hematologic Malignancies (VHM), Relapsed/Refractory Hodgkin Lymphoma (RHL).

Name of Inhibitor	Mechanism of Action	Type of Hematologic Malignancy	Approval Status	Combination Therapies	Clinical Trial Phase	Adverse Effects
Ipilimumab (Yervoy)	Inhibits CTLA-4, leading to enhanced T cell activation and proliferation	RHL	FDA Approved	Combined with nivolumab (PD-1 inhibitor)	III/IV	Fatigue, diarrhea, rash, colitis
Tremelimumab	Inhibits CTLA-4, leading to enhanced T cell activation and proliferation	RHL	Clinical Trials	Combined with durvalumab (PD-L1 inhibitor)	III	Fatigue, nausea, rash, colitis
AGEN1884	Inhibits CTLA-4, enhancing T cell activation and proliferation	VHM	Clinical Trials	Combined with other immunotherapies	I/II	Under investigation
RELA-067	Inhibits CTLA-4, leading to enhanced T cell activation and proliferation	VHM	Clinical Trials	Combined with other immunotherapies	I/II	Under investigation
ONC-392	Inhibits CTLA-4, reducing regulatory T cell suppression and enhancing effector T cell function	VHM	Clinical Trials	Combined with PD-1/PD-L1 inhibitors	I/II	Under investigation
XmAb20717	Bispecific antibody targeting CTLA-4 and PD-1, enhancing T cell activation	VHM	Clinical Trials	Monotherapy and combination with other checkpoint inhibitors	I/II	Under investigation

**Table 11:** Novel approaches in NK- and T cell-based immunotherapies. Abbreviations: Hematologic Malignancies (HM), Solid Tumors (ST), Various Cancers (VC).

Approach	Description	Therapeutic Application	Status
NK Cells + Immune Checkpoint Blockade	Combination of NK cells with checkpoint inhibitors to enhance immune response	VC	Clinical Trials
CAR-NK Cell Therapy	NK cells engineered to express chimeric antigen receptors for targeted cancer cell elimination	HM, ST	Clinical Trials
CAR-T Cell Therapy	T cells engineered to express chimeric antigen receptors for targeted cancer cell elimination	HM, ST	FDA Approved, Clinical Trials
Artificial Adjuvant Vector Cells	Artificial cells designed to enhance NK and T cell activation and targeting	VC	Preclinical/Clinical Trials
NK Cells + Monoclonal Antibodies	NK cells used in conjunction with monoclonal antibodies to target specific cancer cells	HM	Clinical Trials
TCR-Engineered T Cell Therapy	T cells engineered to express specific T cell receptors for precise targeting of cancer antigens	HM, ST	Clinical Trials
NK Cells + Cytokine Therapy	Combination of NK cells with cytokines to boost immune response against cancer cells	VC	Preclinical/Clinical Trials
Bispecific T-Cell Engagers (BiTEs)	Antibodies that simultaneously bind to T cells and cancer cells, bringing them into proximity	HM, ST	FDA Approved, Clinical Trials
NK Cell-Derived Exosomes	Exosomes derived from NK cells used for delivering therapeutic molecules	VC	Preclinical Trials
Trispecific Killer Engager (TriKE)	Molecules that engage NK cells with cancer cells and provide a co-stimulatory signal	HM	Preclinical/Clinical Trials
Dual-Affinity Re-Targeting (DART) molecules	Antibodies designed to bind two different antigens, enhancing immune cell targeting	VC	Preclinical/Clinical Trials

**Table 12:** Roles and actions of macrophages in hematologic malignancies.

Macrophage Function	Action	Effect
Cytokines and Chemokines	Cytokines such as interferons (IFNs), tumor necrosis factor alpha (TNF-α), interleukins (e.g., IL-1, IL-6, IL-12), and chemokines released by other immune cells or produced by macrophages themselves can activate macrophages.	These small molecules bind to specific receptors on macrophages, initiating signaling pathways that induce their activation.
Opsonization	Opsonins, such as antibodies and complement proteins, coat pathogens and enhance their recognition and phagocytosis by macrophages.	Engagement of opsonin receptors on macrophages triggers signaling events that lead to their activation and phagocytic activity.
Phagocytic Receptors	Macrophages express various phagocytic receptors, including Fc receptors and complement receptors, which recognize opsonized pathogens and facilitate their internalization.	Successful binding of these receptors activates downstream signaling pathways that promote phagocytosis and microbial killing.
Inflammatory Mediators	Inflammatory mediators such as prostaglandins, leukotrienes, and reactive oxygen species (ROS) released during inflammation can activate macrophages.	These molecules contribute to the inflammatory response and induce macrophage activation, promoting enhanced anti-tumor activity in hematologic malignancies.
Toll-like receptor (TLR) agonists	TLRs are key molecular sensors that recognize the presence of pathogens and other danger signals.	Stimulation of TLRs on macrophages can lead to their activation and increased ability to eliminate hematologic cancer cells.
Interferons	When used in combination therapy, they can activate macrophages, stimulate the production of chemokines and pro-inflammatory cytokines, and increase the expression of MHC molecules on cancer cells.	This facilitates their recognition by the immune system, resulting in the activation of downstream signaling, deciding the fate of hematologic cancer cells.
CAR-M	Macrophages are engineered to express receptors on their surface, facilitating the recognition of surface antigens either with antibodies or specific ligands present on target cells.	CAR-Ms can phagocytose tumors directly after identifying specific antigens on hematologic cancer cells. Additionally, active CAR-Ms may secrete inflammatory molecules such as IFN-γ, IL-12, and TNF-α to encourage M1 polarization and activate antigen-presenting cells (APCs) in the tumor microenvironment (TME).

**Table 13:** Highlighted strengths and disadvantages of the proposed therapeutic strategy.

Name of Therapy	Potential Benefits	Disadvantages
TIM-3 (T cell immunoglobulin and mucin domain 3)	Combined PD-1/PD-L1 with TIM-3/Gal-9 blockade could prevent CD8^+^ T-cell exhaustion in advanced AML [85]. PD-1 combined with TIM-3 blockades could stimulate potential anti-tumor T cell responses in melanoma [86]. In xenograft models, anti-TIM-3 IgG2a antibody could improve cytotoxic activities and eradicate AML leukemic stem cells [87].	Lack of valid biomarkers which can predict successful treatment with this combination [88]. Combinations will have to be patient-tailored since they are likely to be more toxic than single agents and more expensive. Cells usually have functionally redundant pathways which could override and compensate for each other [89].
TIGIT	TIGIT suppresses both innate and adaptive immunity by a variety of mechanisms, such as initiating T/NK cell-intrinsic inhibition, producing immunosuppressive DCs, blocking CD226 signaling, boosting Treg immunosuppression, and encouraging Fap2-induced T/NK cell inhibition [90].	There is currently no reliable biomarker for anti-TIGIT therapy. As a result, future studies should concentrate on identifying new biomarkers or targeting TIGIT using alternative strategies, such as CAR-T cells, antibody-drug conjugates, and bispecific antibodies [91].
Small molecule inhibitors	Easier cellular entry, oral effectiveness, and comparatively cost-efficient synthesis [8]. In vivo studies indicate that P5091 is well tolerated, inhibits malignant cell growth, and extends survival [23].	Pulmonary toxicity in preclinical studies [19]. Studies on biochemical and cellular characterization of lead compounds, in addition to extensive PK, pharmacodynamics, and toxicology studies, are required [92].
PI3K inhibitors	Several inhibitors passed clinical trials and are approved by FDA. Demonstrated desired therapeutic effects on various cancers. Several inhibitor alternatives available in market [93].	Adverse effects remain major concern for this therapy. On-target toxicities severely limit the development of PI3K inhibitors [93].
NFkB inhibitors	Inhibits NF-κB activation by sup-pressing phosphorylation and degradation of IκBα, preventing NF-κB translocation [47]. Inhibits NF-κB activation by targeting the IκB kinase complex and preventing phosphorylation of IκBα [94].	Mild gastro-intestinal symptoms [47]. Cytotoxicity at high doses [94].
CD47 Inhibitors	This therapy aims to synergistically boost the immune system’s ability to target and eliminate cancer cells, while also overcoming resistance mechanisms that tumors may develop [52].	These limitations include resistance mechanisms, toxicity, lack of predictive biomarkers, inadequate effectiveness as a monotherapy, and production difficulties [95].
Neddylation Inhibitors	Widespread antiproliferative activity in cancer cell lines and patient-derived tumor cells, making it a promising agent for hematologic tumors [57]. Currently under phase II/III clinical trials for anti-tumor treatment and shows good safety and tolerability, indicating its good development prospects [96].	Drug resistance is a major challenge [96].

**Table 14:** Summary of combination therapies for hematologic malignancies.

Combination Therapy	Components	Mechanism of Action	Potential Benefits	Clinical Status
PI3K Inhibitor + Proteasome Inhibitor	Idelalisib + Bortezomib	PI3K inhibitors impede signaling pathways regulating cell growth; proteasome inhibitors block protein degradation	Synergistic anti-cancer effects, reduced cell proliferation, enhanced apoptosis	Clinical Trials
PI3K Inhibitor + Immunological Checkpoint Inhibitor	Idelalisib + TIM-3/TIGIT inhibitors	PI3K inhibitors reduce immunosuppression; checkpoint inhibitors enhance T cell activation	Enhanced immune response, suppressed tumor cell proliferation	Preclinical/Clinical Trials
Immunological Checkpoint Inhibitor + Proteasome Inhibitor	PD-1/CTLA-4 inhibitors + Bortezomib	Checkpoint inhibitors boost immune response; proteasome inhibitors regulate apoptosis-controlling protein expression	Enhanced tumor cell apoptosis, boosted immune response	Clinical Trials
NF-κB Inhibitor + TIGIT Inhibitor	NF-κB inhibitors + TIGIT inhibitors	NF-κB inhibitors regulate signaling pathways; TIGIT inhibitors reduce Treg-mediated immunosuppression	Reduced tumor cell proliferation, heightened immune response	Preclinical
NF-κB Inhibitor + Monoclonal Antibody Therapy	NF-κB inhibitors + Monoclonal antibodies	NF-κB inhibitors block survival pathways; monoclonal antibodies target cancer cell receptors	Synergistic anti-tumor impact, enhanced immune-mediated tumor eradication	Preclinical/Clinical Trials
Neddylation Inhibitor + Tumorigenesis Inhibitor	MLN4924 + Tumorigenesis inhibitors	Neddylation inhibitors block protein degradation; tumorigenesis inhibitors impede growth and proliferation processes	Enhanced tumor cell apoptosis, restrained tumor growth and metastasis	Preclinical
Neddylation Inhibitor + Antigen Complex Therapy	MLN4924 + Antigen complex therapy	Neddylation inhibitors prevent protein degradation; antigen complex therapy elicits immune response	Increased tumor cell apoptosis, mounted immune response	Preclinical
CAR-T Cell Therapy + Immunological Checkpoint Inhibitor	CAR-T cells + Pembrolizumab	CAR-T cells target and eliminate cancer cells; checkpoint inhibitors enhance CAR-T cell persistence and function	Augmented CAR-T cell efficacy, enhanced immune response	Clinical Trials
CAR-T Cell Therapy + Radiotherapy	CAR-T cells + Radiotherapy	CAR-T cells target cancer cells; radiotherapy enhances tumor cell destruction	Improved anti-cancer immune response, enhanced tumor cell destruction	Clinical Trials
CAR-T Cell Therapy + Immunomodulatory Drugs	CAR-T cells + Immunomodulatory drugs	CAR-T cells target tumor cells; immunomodulatory drugs boost T cell proliferation and persistence	Effective tumor eradication, enhanced cytokine production	Clinical Trials
Signaling Cascade Inhibitors + Immune Checkpoint Inhibitors	Ibrutinib + Anti-PD-1/PD-L1 antibodies	Signaling inhibitors regulate growth pathways; checkpoint inhibitors boost immune response	Overcome resistance, enhanced anti-tumor immune response	Clinical Trials
CD47 Inhibitor + CAR-T Therapy	Hu5F9-G4 + CAR-T cells	CD47 inhibitors increase phagocytosis of cancer cells; CAR-T cells target and eliminate cancer cells	Enhanced phagocytosis, robust immune response	Clinical Trials
NF-κB Inhibitor + Hyperthermia Therapy	NF-κB inhibitors + Hyperthermia	NF-κB inhibitors block survival pathways; hyperthermia increases treatment sensitivity	Increased apoptosis, enhanced treatment efficacy	Preclinical

**Table 15:** Summary of Other Potential Combination Therapies for Hematologic Malignancies.

Combination Therapy	Components	Mechanism of Action	Potential Benefits	Clinical Status
CAR-T Therapy + Oncolytic Virus Therapy	CAR-T cells + Oncolytic viruses	Enhanced CAR-T cell infiltration and activity, direct oncolytic effects	Increased CAR-T cell efficacy, enhanced immune response	Preclinical/ Clinical Trials
Epigenetic Modifiers + Immunotherapy	Epigenetic drugs + CAR-T cells/checkpoint inhibitors	Improved tumor antigen expression, enhanced immune recognition and response	Improved immune response, enhanced tumor antigen presentation	Preclinical/ Clinical Trials
Metabolic Inhibitors + Immune Checkpoint Inhibitors	Metabolic inhibitors + Checkpoint inhibitors	Disrupted cancer cell metabolism, reduced tumor growth, enhanced immune response	Enhanced antitumor response, reduced tumor growth	Preclinical/ Clinical Trials
PARP Inhibitors + Immunotherapy	PARP inhibitors + CAR-T cells/checkpoint inhibitors	Increased DNA damage, improved immune recognition, and response	Enhanced tumor cell death, improved immune response	Preclinical/ Clinical Trials
Autophagy Inhibitors + Chemotherapy	Autophagy inhibitors + Chemotherapy	Increased chemotherapy efficacy, reduced cancer cell survival	Enhanced chemotherapy effects, reduced tumor cell survival	Preclinical/ Clinical Trials
Bcl-2 Inhibitors + Immunotherapy	Bcl-2 inhibitors + CAR-T cells/checkpoint inhibitors	Increased tumor cell apoptosis, enhanced immune response	Improved tumor cell death, enhanced immune response	Preclinical/ Clinical Trials
Proteasome Inhibitors + Histone Deacetylase Inhibitors	Proteasome inhibitors + Histone deacetylase inhibitors	Synergistic induction of apoptosis, improved tumor cell death	Enhanced apoptosis, improved tumor cell death	Preclinical/ Clinical Trials
Checkpoint Inhibitors + TLR Agonists	Checkpoint inhibitors + TLR agonists	Enhanced activation of innate and adaptive immune responses, improved antitumor activity	Improved immune response, enhanced tumor destruction	Preclinical/ Clinical Trials
Angiogenesis Inhibitors + Immune Checkpoint Inhibitors	Angiogenesis inhibitors + Checkpoint inhibitors	Reduced tumor vascularization, enhanced immune response	Reduced tumor growth, improved immune cell infiltration	Preclinical/ Clinical Trials
Anti-CD47 Therapy + Radiotherapy	Anti-CD47 antibodies + Radiotherapy	Enhanced phagocytosis, improved immune response, increased tumor cell death	Improved tumor clearance, enhanced immune response	Preclinical/ Clinical Trials

**Table 16:** Failed Clinical Trials in Hematologic Malignancies. Acute Myeloid Leukemia (AML), Breast Cancer (BC), B Cell Malignancies (BCM), Chronic Lymphocytic Leukemia (CLL), Chronic Myelo-Monocytic Leukemia (CMML), Gastric cancer (GC), Hematologic Malignancies (HM), Multiple Myeloma (MM), Non-Small Cell Lung Cancer (NSCLC); Mantle Cell Lymphoma (MCL), Refractory Hematologic Malignancies (RHM).

Therapy	Target Disease	Combination	Reason for Failure
Orelabrutinib	BCM	-	Significant safety concerns
Nemtabrutinib (formerly ARQ 531)	CLL and MCL	-	Efficacy and safety issues
TTI-621	Relapsed or RHM	-	Significant safety issues
Hu8F4	AML and CMML	-	Limited efficacy and significant toxicity
Ivosidenib and Venetoclax with or without Azacitidine	IDH1-mutated HM	Combination of Ivosidenib and Venetoclax, sometimes with Azacitidine	Safety challenges and unmet therapeutic outcomes
Afuresertib and Fulvestrant	HR+/HER2 BC (with implications for HM)	Combination of Afuresertib and Fulvestrant	Insufficient efficacy in Phase III trials
Rilotumumab	GC and HM	-	Safety concerns and lack of efficacy in Phase III trials
Bavituximab	NSCLC and HM	-	Lack of efficacy in Phase III trials
Selinexor	MM and other HM	-	Significant toxicity and limited efficacy in later-stage trials

**Table 17:** Future actions and direction lines for combinational therapies.

Future Actions and Direction Lines for Combinational Therapies	
Targeted Combinations	As genomic profiling becomes more sophisticated, therapies will increasingly be tailored to the genetic and molecular characteristics of individual patients’ tumors. This approach will enable the selection of drug combinations that target specific mutations, pathways, or microenvironmental factors driving the malignancy [142,143].
Predictive Biomarkers	The identification and validation of biomarkers that predict response to specific drug combinations will play a crucial role in personalizing therapy. For example, using biomarkers to guide the use of immunotherapy combinations with targeted therapies could optimize treatment efficacy and minimize toxicity [144].
Immunotherapy Combinations	CAR-T cells with immune checkpoint inhibitors: CAR-T therapy has shown remarkable success in some hematologic malignancies, but resistance and relapse remain challenges. Combining CAR-T cells with immune checkpoint inhibitors (e.g., anti-PD-1/PD-L1 or anti-CTLA-4) could enhance the persistence and efficacy of CAR-T cells by overcoming the immunosuppressive tumor microenvironment [142,143].
Bispecific Antibodies and Cytokines	The use of bispecific antibodies that target both the cancer cells and immune cells, combined with cytokine therapies to boost the immune response, is a promising strategy. These combinations aim to enhance the immune system’s ability to recognize and eliminate cancer cells more effectively [145].
Stromal and Immune Modulators	The tumor microenvironment, including stromal cells, immune cells, and the extracellular matrix, plays a critical role in the progression and resistance of hematologic malignancies. Combinational therapies that target both the cancer cells and their supportive microenvironment could prevent resistance and improve outcomes [146].
Hypoxia-Targeted Therapies	Targeting hypoxia-inducible factors (HIFs) in the tumor microenvironment, in combination with other therapies, could reduce the adaptation of cancer cells to hypoxic conditions, which is often associated with resistance to therapy [147].
Epigenetic Modifiers with Chemotherapy	Combining epigenetic therapies, such as DNA methyltransferase inhibitors or histone deacetylase inhibitors with standard chemotherapy could enhance the sensitivity of cancer cells to treatment. Epigenetic modifications often drive resistance, so targeting these changes could overcome resistance mechanisms [148].
Combining Epigenetic and Immunotherapies	There is growing interest in combining epigenetic drugs with immunotherapies to increase the immunogenicity of tumors. For example, epigenetic drugs could upregulate the expression of antigens or immune-related genes, making the cancer cells more susceptible to immune attack [148].
Next-Generation Targeted Therapies	The development of next-generation small molecule inhibitors that target previously “undruggable” proteins or that have greater specificity and potency is a major focus. These could be used in combination with existing therapies to enhance efficacy and reduce side effects [149].
Synthetic Lethality Approaches	Combining drugs that exploit synthetic lethality—where the simultaneous inhibition of two genes or pathways leads to cancer cell death, but inhibition of either alone does not—could provide a powerful strategy against hematologic malignancies with specific genetic alterations [150].
Sequential and Adaptive Combinations	Instead of static combination regimens, future therapies might involve adaptive or sequential combinations, where treatments are adjusted based on the real-time monitoring of tumor evolution and resistance patterns. This dynamic approach could help prevent the emergence of drug-resistant clones.
Dual-Targeting Strategies	Combining two or more drugs that target different aspects of the same pathway or cellular process could prevent the cancer from developing resistance through alternative pathways [145].
Big Data for Predictive Modeling	Integrating data from genomics, proteomics, and patient outcomes into predictive models can help forecast which combinations will be most effective for specific patient populations. This approach could lead to the rapid identification of novel combinations that might not have been considered through traditional research methods [151].
Targeted Delivery Systems	Advances in drug delivery technologies, such as nanoparticles or conjugated antibodies, could allow for more precise targeting of drug combinations to cancer cells while sparing healthy tissues. This approach could minimize side effects and improve patients’ quality of life during treatment [152].
Reducing Off-Target Effects	Combining therapies that have complementary mechanisms of action but non-overlapping toxicity profiles could reduce the cumulative side effects experienced by patients, making long-term treatment more tolerable [153].
Master Protocols	Future clinical trials for combinational therapies are likely to involve master protocols where multiple therapies are tested simultaneously across different subtypes of hematologic malignancies. This approach can accelerate the identification of effective combinations [142,143,144].

## Data Availability

Data sharing is not applicable to this article.
